# Prediction-Aided Adaptive Semantic Communication for IoT Sensor Streams Under Outdated Channel State Information

**DOI:** 10.3390/s26196124

**Published:** 2026-09-27

**Authors:** Osman Kaya, Muhammet Ali Karabulut, Can Eyüpoğlu, Oktay Karakuş

**Affiliations:** 1 Department of Electronics Engineering, Turkish Air Force Academy, National Defence University, 34149 Istanbul, Türkiye; osman.kaya2@msu.edu.tr (O.K.); muhammetali.karabulut@msu.edu.tr (M.A.K.); 2 Department of Computer Science and Informatics, Cardiff University, Cardiff CF24 4AG, UK; eyupogluc@cardiff.ac.uk or can.eyupoglu@msu.edu.tr; 3 Department of Computer Engineering, Turkish Air Force Academy, National Defence University, 34149 Istanbul, Türkiye

**Keywords:** semantic communication, joint source-channel coding, channel prediction, outdated channel state information, channel aging, probabilistic forecasting, measured 5G channel traces, internet of things

## Abstract

Adaptive semantic communication can save radio resources by matching the representation rate to channel quality, yet its decisions become unreliable when channel state information reaches the transmitter with delay. This study examines that overlooked failure mode and develops a prediction-aided framework for rate-adaptive transmission of Internet of Things sensor streams. A compact long short-term memory network forecasts a distribution of the receive signal-to-noise ratio (SNR), and a risk-aware controller selects the smallest rate whose expected semantic distortion under that forecast meets a fidelity target. The controller is independent of the semantic codec and accounts for forecast uncertainty rather than relying on a single predicted value. The framework is evaluated on six real sensor streams, over time-correlated fading channels and on SNR logs measured in a commercial 5G/LTE network, with all schemes held to the same average bandwidth and with confidence intervals over independent channel realizations. Adaptation based on stale channel information loses its advantage over fixed-rate transmission as mobility or feedback delay increases, both in simulation and on measured logs. Channel prediction recovers a substantial part of this loss, a single predictor serves the whole mobility range without retraining, and the learned forecast remains reliable when the channel statistics assumed by a model-based predictor are wrong. Prediction also keeps its advantage over stale channel information under drifting mobility, varying channel-estimation quality and frequency-selective multipath, and a predictor trained for one mobility regime can be adapted to another within seconds. A second-order analysis, whose assumptions are verified on the trained codec, explains how forecast uncertainty and the curvature of the codec’s distortion response affect the rate decision, including how often uncertainty changes the choice among discrete rates. The resulting framework is lightweight and suitable for practical semantic links operating with delayed feedback.

## 1. Introduction

Semantic and goal-oriented communication reorganizes the transmission problem around the meaning that the receiver needs rather than the bits that the transmitter happens to hold [1,2,3]. In the deep learning instantiation of this idea, an encoder and a decoder are trained jointly across a channel model, so that the reconstruction quality degrades gracefully as the channel worsens instead of collapsing abruptly [4,5]. This end-to-end view grew out of learning-based physical-layer design, in which the transmitter and the receiver are represented by a single autoencoder trained through the channel [6] and which has been demonstrated over the air [7]. The same principle has reached multiple access: deep autoencoders learn sparse code multiple access (SCMA) codebooks and decoders jointly [8], and combining the autoencoder with a Wasserstein generative adversarial network makes end-to-end SCMA encoding and decoding more robust to channel noise [9]. Semantic communication carries this idea from symbols to meaning. For massively deployed Internet of Things (IoT) sensing, where the uplink is narrow, the devices are cheap and the data are long time series whose information content changes slowly, this reorganization is particularly attractive: what a monitoring back end needs from a vibration or temperature stream is a decision, not a faithful waveform [10,11].

A recurring design element in this literature is adaptivity. Because the wireless channel varies, a fixed compression rate may waste resources in good conditions and provide insufficient protection in poor ones. Attention-based adaptation to the signal-to-noise ratio (SNR) [12], variable-length and rate-adaptive semantic coding [13], and feedback-aided schemes [14] all condition the encoder, the decoder, or the number of transmitted symbols on channel quality. Most reported gains are evaluated with contemporaneous or perfectly matched SNR information, so the delay between channel measurement and transmission is not part of the adaptation problem.

This simplification becomes important in a practical link. The transmitter learns the channel through pilots and feedback, so its channel state information (CSI) is delayed by an interval τ that includes estimation, feedback and processing. During that interval the channel decorrelates, a phenomenon known as channel aging [15], and the mismatch grows with mobility. Classical link adaptation faced exactly this issue and answered it with fading prediction [16,17]. The semantic communication literature has nevertheless retained the perfect-CSI idealization in many adaptive designs. The closest related scheme, the predictive and adaptive deep coding (PADC) framework of Zhang et al. [18], predicts the quality that an image will reach for a given compression ratio and channel SNR and then selects the smallest ratio that satisfies a quality constraint. PADC therefore predicts the transmission quality for a known SNR. It does not address how the rate decision should behave when the SNR itself is outdated, or how the uncertainty of an SNR forecast should enter that decision.

This article therefore treats outdated CSI as the central design constraint. Three observations motivate the approach. First, stale CSI can erase the gain of rate adaptation and can even make an adaptive link less reliable than a bandwidth-matched fixed-rate link. Second, the measured relation between channel quality and semantic distortion is predominantly convex over the SNR range where rate decisions are made, so an optimistic forecast error is more costly than a pessimistic error of the same magnitude. A point forecast can therefore produce an optimistic rate decision even when its average error is small. Third, a calibrated predictive distribution allows the controller to average over that uncertainty without changing the semantic codec.

The main contributions are as follows.

- We formulate rate-adaptive semantic communication with delayed, noisy CSI and define the semantic adaptation mismatch between decisions based on stale and current channel information. The experiments reveal a clear mobility- and delay-dependent regime in which outdated-CSI adaptation performs worse than a fixed-rate link at the same average bandwidth. To the best of our knowledge, this failure mode has not previously been quantified for semantic rate control.
- We propose a prediction-aided framework in which a long short-term memory (LSTM) network [19] forecasts the receive SNR over the horizon τ and emits quantiles trained with the pinball loss [20,21], whose calibration is verified on held-out channel realizations and measured logs. A risk-aware controller selects the smallest rate whose belief-averaged distortion, that is, the Bayes risk under the predictive law [22], meets a semantic fidelity target. Unlike point-prediction approaches, the controller uses the entire estimated uncertainty and remains independent of the codec architecture.
- We derive a second-order relation (Proposition 1) between the prediction error variance $\sigma_{e}^{2}$ and the excess semantic distortion incurred by plug-in control, together with a condition (Corollary 1) under which prediction reduces the leading-order loss. A second result (Proposition 2) carries the analysis to the discrete rate set and shows when forecast uncertainty moves the decision to the next rate. The regularity and curvature assumptions behind these results are stated explicitly and checked on the measured distortion surface of the trained codec. This analysis explains why uncertainty matters most when the forecast is difficult and the distortion surface is strongly curved.
- We evaluate the framework on six real sensor streams from the Numenta Anomaly Benchmark [23,24] and on measured SNR logs recorded by handsets in a commercial 5G/LTE network under driving and static conditions [25]. To the best of our knowledge, this is the first study of semantic rate control under outdated CSI that is driven by measured channel traces together with real sensor data, rather than by simulation alone. The comparison includes six baselines, among them a genie-aided Wiener predictor that is given the exact Clarke autocorrelation, and every scheme is tuned to exactly the same average bandwidth. All results carry 95% confidence intervals and paired significance tests. We further report results for each sensor stream, for a single predictor that serves the whole mobility range without retraining, for different quantile representations and for downstream monitoring tasks. The evaluation extends to non-stationary Doppler, time-varying CSI quality and frequency-selective multipath, and it measures the cost of adapting a trained predictor to a new regime instead of training it again. Two model-mismatch regimes show that the genie-aided model-based predictor retains a modest edge only when its assumptions hold.

Table 1 summarizes how these elements relate to the closest prior work.

The remainder of the paper is organized as follows. Section 2 reviews related work. Section 3 states the system model and the rate-control problem. Section 4 develops the proposed framework. Section 5 contains the performance analysis. Section 6 and Section 7 describe the experimental setup and the results. Section 8 concludes.

## 2. Related Work

Deep joint source–channel coding (JSCC) [4] replaces the separate source and channel coding chain with a pair of trained networks and removes the cliff effect of digital schemes. DeepSC [5] extended the idea to text with a sentence-level semantic similarity objective, and the survey literature [3,28] now treats deep JSCC as the canonical realization of semantic transmission, with nonlinear transform coding [29] refining the basic construction. The same learned design has since been carried to speech [30], to task-oriented transmission for several users sharing a channel [31] and to multi-antenna links [32]. Adaptivity was added soon after: ADJSCC [12] inserts attention blocks that modulate the latent according to the SNR, so that a single model covers a range of channel conditions; DeepJSCC-f [14] exploits a feedback link to refine the transmission over several passes; bandwidth-agile deep JSCC [26] produces successively refinable representations; and rate-adaptive mechanisms [13] vary the number of transmitted symbols with the channel and the content. PADC [18] adds an oracle network that predicts the reconstruction quality for a given content, SNR and compression ratio, and uses it to choose the smallest ratio meeting a quality target. In all of these designs the SNR that conditions the encoder or the rate decision is treated as contemporaneous with the transmitted block. Our work retains this codec family but makes the acquisition delay and uncertainty of that conditioning information explicit.

A second line of work, channel prediction, has long studied the loss of CSI accuracy over time. Truong and Heath [15] formalized channel aging for massive multiple-input multiple-output (MIMO) systems, and adaptive-modulation studies had earlier shown that outdated fading estimates erode the gains of link adaptation [16]. Long-range fading prediction has therefore been studied for decades, from linear autoregressive and Wiener predictors [17] to neural predictors [33,34] and, more recently, attention-based architectures [27]. This literature treats prediction as a signal-processing problem judged by the mean-squared error of $\hat{h}$; it does not ask how that error maps into an application-level distortion. Our work addresses this link: we treat the predictor as one component of the system and study the semantic distortion that its errors cause. Because this distortion depends on the whole predictive law, we also rely on tools from probabilistic forecasting: quantile regression [20], proper scoring rules for assessing predictive distributions [35], and split-sample recalibration of predicted quantiles in the spirit of conformalized quantile regression [36], which we examine as an optional step.

A third line of work concerns the IoT setting itself. For networked sensing, the value of a transmission is unevenly distributed over time. Age of information [37] and the data-significance view of Uysal et al. [10] formalize this, and goal-oriented IoT designs [11] allocate resources according to the usefulness of the data for a downstream goal. These works focus on the value of the content, whereas the present work addresses a complementary question: how to adapt the transmission rate to a channel that is known only through delayed feedback.

Among these designs, PADC [18] is the closest to ours, since it also couples a prediction module to a rate-adaptive semantic codec. Three differences define our contribution. The first is what is predicted: PADC predicts the reconstruction quality for a given SNR, which is assumed known when the rate is chosen, whereas we predict the future SNR itself from delayed and noisy CSI and study feedback age explicitly across mobility and delay regimes, for IoT sensor streams rather than images. The second is the use of a belief instead of a point estimate: we show that plugging a point forecast into a convex distortion map introduces a systematic bias, and we replace it with a calibrated predictive distribution; the ablation between our point and distributional variants isolates this effect and shows where it matters. The third concerns the analysis and the evidence: we give a second-order characterization of how prediction error becomes semantic distortion, which yields an explicit condition for when prediction pays, and we test the framework on measured 5G channel logs in addition to fading models. Table 1 lists these differences together with the other adaptive designs discussed above.

## 3. System Model and Problem Formulation

### 3.1. Source and Rate-Adaptive Semantic Codec

A sensor node observes a scalar stream and groups it into blocks $x_{n}\in R^{L}$ of *L* consecutive samples, indexed by the block time *n*. A semantic encoder $f_{\theta }$ maps a block to a latent vector

(1) $$z_{n}=f_{\theta }\left(x_{n},k_{n},{\hat{\gamma }}_{n}\right)\in R^{d},\;d=2\max K,$$

where $k_{n}$ is the number of complex channel uses allocated to the block and ${\hat{\gamma }}_{n}$ is the transmitter’s forecast of the receive SNR (the predictive median ${\hat{\gamma }}_{n}^{\left(0.5\right)}$ in the proposed scheme). The latent is nested: the first 2k real coordinates form a self-contained description for every admissible k∈K={2,4,…,16}, so a single trained model realizes the whole rate ladder without retraining, in the spirit of bandwidth-agile deep JSCC [26]. Writing $m_{k}$ for the truncation mask, the transmitted symbol vector is

(2) $$s_{n}=\frac{m_{k_{n}}⊙z_{n}}{p_{n}},\;p_{n}=\frac{∥m_{k_{n}}⊙z_{n}∥}{\sqrt{2k_{n}}},\;\frac{1}{2k_{n}}{∥s_{n}∥}^{2}=1,$$

so that the power per transmitted symbol is exactly unity at every rate and $p_{n}$ carries the block amplitude. The bandwidth ratio of the link is $k_{n}/L$.

At the receiver, coherent detection and equalization reduce the block to an equivalent additive white Gaussian noise (AWGN) observation

(3) $$y_{n}=s_{n}+w_{n},\;w_{n}∼N\;\left(0,\gamma_{n}^{-1}I\right),$$

with $\gamma_{n}$ the instantaneous receive SNR defined below. The semantic decoder $g_{\phi }$ reconstructs ${\hat{x}}_{n}$ from (3) as ${\hat{x}}_{n}=g_{\phi }\left(y_{n},k_{n},\gamma_{n},p_{n}\right)$, where $p_{n}$ is the block power normalizer of (2), transmitted as a protected scalar header. The per-block normalized squared error is

(4) $$d_{n}=\frac{{∥{\hat{x}}_{n}-x_{n}∥}^{2}}{{∥x_{n}∥}^{2}+\epsilon },$$

where ϵ prevents division by zero. The controller uses the energy-weighted aggregate normalized mean-squared error (NMSE)

(5) $$D\left(k,\gamma \right)=\frac{E[{∥\hat{x}-x∥}^{2}|k,\gamma ]}{E[{∥x∥}^{2}]},$$

which is measured once, offline, on the validation split and stored as a small lookup table; its construction is detailed in Section 4.4. For reporting, the per-block value $d_{n}$ is additionally saturated at one, so that rare blocks with almost zero energy cannot dominate the mean.

### 3.2. Time-Varying Channel and CSI Aging

The channel gain $h_{n}$ is a zero-mean complex Gaussian process with $E|h_{n}{|}^{2}=1$ and a Clarke–Doppler spectrum [38,39], so that

(6) $$E\;\left[h_{n}h_{n-m}^{*}\right]=J_{0}\left(2\pi f_{D}Tm\right),$$

where $J_{0}$ is the zeroth-order Bessel function of the first kind, $f_{D}$ is the maximum Doppler frequency and *T* is the block duration; $f_{D}T$ is the normalized Doppler that parameterizes mobility throughout the paper. The instantaneous receive SNR is

(7) $$\gamma_{n}={\left|h_{n}\right|}^{2}\rho ,$$

with ρ being the average SNR. It is convenient to work in the logarithmic domain and we write γ for $10\log_{10}{\left|h_{n}\right|}^{2}+10\log_{10}\rho$ in dB whenever distortion is evaluated. The fading model is used for the controlled mobility and delay sweeps; Section 7.8 replaces it with SNR logs measured in a commercial 5G/LTE network, for which no model of $h_{n}$ is assumed.

The transmitter does not observe $h_{n}$. It receives, over a feedback link, a noisy estimate delayed by τ blocks,

(8) $${\hat{h}}_{n-\tau }=h_{n-\tau }+e_{n-\tau },\;e_{n-\tau }∼\mathrm{CN}\left(0,\sigma_{\mathrm{csi}}^{2}\right),$$

and it holds a window of *W* such observations, $O_{n}=\left\{{\hat{h}}_{n-\tau },\ldots ,{\hat{h}}_{n-\tau -W+1}\right\}$. Combining (6) and (8), the mean-squared error of using the newest observation directly as a surrogate for $h_{n}$ is

(9) $$E|h_{n}-{\hat{h}}_{n-\tau }{|}^{2}=2\left(1-J_{0}\left(2\pi f_{D}T\tau \right)\right)+\sigma_{\mathrm{csi}}^{2},$$

which grows quadratically in $f_{D}T\tau$ for small arguments. Equation (9) is the quantitative statement of channel aging that motivates this work, and it is the quantity that a predictor is meant to reduce.

### 3.3. Rate Control Problem

Let ${\hat{F}}_{n}$ denote the predictive law that the transmitter holds for $\gamma_{n}$ given $O_{n}$. The link operates under a semantic fidelity target $D_{t}$ and spends as little bandwidth as possible to meet it:

(10) $$k_{n}^{★}=\min\left\{k\in K\;:\;E_{\gamma ∼{\hat{F}}_{n}}\;\left[D(k,\gamma )\right]\leq D_{t}\right\},$$

with $k_{n}^{★}=\max K$ when the set is empty.

We use the word risk in its decision-theoretic sense: for a candidate rate *k*, the quantity $E_{\gamma ∼{\hat{F}}_{n}}\left[D\left(k,\gamma \right)\right]$ in (10) is the posterior expected loss, or Bayes risk, of that decision under the transmitter’s predictive law [22]. The controller is risk-aware because it constrains this expected distortion instead of the distortion evaluated at a single predicted SNR. It does not impose a probabilistic constraint on the semantic outage defined below. A direct outage constraint of the form ${\mathrm{Pr}}_{{\hat{F}}_{n}}\left\{D\left(k,\gamma \right)>D_{t}\right\}\leq \varepsilon$ is a different design; because D(k,·) is non-increasing in γ, it reduces to the quantile rule

(11) $$k_{n}^{\left(\varepsilon \right)}=\min\left\{k\in K\;:\;D\left(k,{\hat{\gamma }}_{n}^{\left(\varepsilon \right)}\right)\leq D_{t}\right\},$$

where ${\hat{\gamma }}_{n}^{\left(\varepsilon \right)}$ is the ε-quantile of ${\hat{F}}_{n}$. Rule (11) is evaluated as a variant in Section 7.11.

Three quantities are reported: the average bandwidth $\bar{k}=E\left[k_{n}\right]$, the saturated mean distortion $\bar{D}=E\left[\min\left(d_{n},1\right)\right]$, and the semantic outage probability

(12) $$P_{\mathrm{out}}=\mathrm{Pr}\left\{d_{n}>D_{\mathrm{req}}\right\},$$

where $D_{\mathrm{req}}$ is an application-level fidelity requirement that is common to all schemes. The controller target $D_{t}$ in (10) is a tuning parameter of each scheme, whereas $D_{\mathrm{req}}$ is fixed by the application; keeping the two separate ensures that outage is measured against the same requirement for every scheme. Unless stated otherwise $D_{\mathrm{req}}=0.25$, and results for other values are given in Section 7.4. Because different schemes reach different $\left(\bar{k},\bar{D}\right)$ pairs for the same $D_{t}$, all comparisons in Section 7 are made at a common average bandwidth, obtained by tuning $D_{t}$ for each scheme as described in Section 6.5.

The schemes compared in this paper (Section 6) differ only in ${\hat{F}}_{n}$. A plug-in scheme uses a point forecast, ${\hat{F}}_{n}=\delta_{{\hat{\gamma }}_{n}}$, so that (10) reduces to $D\left(k,{\hat{\gamma }}_{n}\right)\leq D_{t}$. A belief scheme uses a non-degenerate law. The genuine-CSI reference uses ${\hat{F}}_{n}=\delta_{\gamma_{n}}$, which is unattainable in practice and indicates how much a perfect forecast could gain.

## 4. Proposed Framework

Figure 1 shows the architecture. Three elements are added to a conventional rate-adaptive semantic link: an LSTM channel predictor with a quantile head, a risk-aware rate controller, and the feedback of the predictive median into the encoder conditioning of (1).

### 4.1. LSTM Channel Predictor with a Quantile Head

The predictor consumes the window $O_{n}$ represented as a sequence of triples $\left(ℜ\hat{h},\;ℑ\hat{h},\;\log_{10}{\left|\hat{h}\right|}^{2}\right)$, an invertible reparameterization of exactly the information available to the model-based baseline, so that the comparison isolates the estimator and not the input. A two-layer LSTM [19] with 64 hidden units produces a feature vector that feeds two linear heads: a point head trained with the squared error, and a quantile head producing Q=9 levels q∈{0.05,0.15,0.25,0.35,0.50,0.65,0.75,0.85,0.95} trained with the pinball loss [20],

(13) $$L_{\mathrm{pin}}=\frac{1}{Q}\sum_{i=1}^{Q}\max\left(q_{i}\left(\gamma -{\hat{\gamma }}^{\left(q_{i}\right)}\right),\;\left(q_{i}-1\right)\left(\gamma -{\hat{\gamma }}^{\left(q_{i}\right)}\right)\right),$$

with the head outputs sorted so that the quantiles are monotone by construction. Multi-horizon quantile recurrent forecasting of this kind is standard in probabilistic time-series prediction [21,40]; what is new here is its use as the belief that drives a semantic rate controller. Quantile regression is preferred over sampling-based uncertainty such as Monte Carlo dropout [41] because it yields the predictive law in a single forward pass, which matters when the whole computation must fit inside the coherence time. The total training objective is $L=L_{\mathrm{mse}}+3L_{\mathrm{pin}}$, with $L_{\mathrm{pin}}$ as in (13). The network is not given $f_{D}$, $\sigma_{\mathrm{csi}}^{2}$ or the shape of the Doppler spectrum.

The choice of quantiles follows from what the controller needs, namely the expectation in (10). Writing it through the quantile function $F^{-1}$ of the predictive law gives $E\left[D\left(k,\gamma \right)\right]=\int_{0}^{1}D\left(k,F^{-1}\left(u\right)\right)\;du$, so a set of predicted quantiles is exactly the information required by a quadrature rule for this integral, without committing to a parametric family for the SNR forecast error. A small number of levels is enough to capture the relevant part of the law, because D(k,·) is smooth over the operating region and saturates at high SNR. Nine levels place four points in each half of the law and include the 5% and 95% levels used for the 90% predictive interval. The number and placement of the levels are examined in Section 7.11.

### 4.2. Calibration Check and Optional Recalibration

Pinball training makes each output a consistent estimate of its quantile on the training distribution, but finite training data, early stopping and a shift between training and deployment can leave coverage errors. Before a predictor is used, its coverage is therefore checked on a separate set of calibration data that was used neither for fitting nor for checkpoint selection. The same set allows a simple recalibration in the spirit of conformalized quantile regression [36]: for every level $q_{i}$ an offset $c_{i}$ is set to the empirical $q_{i}$-quantile of the residuals $\gamma -{\hat{\gamma }}^{\left(q_{i}\right)}$, and the controller would use ${\hat{\gamma }}^{\left(q_{i}\right)}+c_{i}$, re-sorted to remain monotone. We evaluate this step as an option in Section 7.1 and Section 7.11. In our experiments the pinball-trained head was well calibrated without it, and on measured logs a calibration set drawn from other recordings increased the coverage error. The proposed scheme therefore uses the quantile head directly, and the offsets are reported only as an ablation.

### 4.3. Risk-Aware Rate Control

The controller represents uncertainty by placing the mass $w_{i}$ on the *i*-th predicted quantile value, where $w_{i}$ is the width of the probability interval that the level represents; i.e., $w_{i}=\frac{1}{2}\left(q_{i+1}-q_{i-1}\right)$ with $q_{0}=-q_{1}$ and $q_{Q+1}=2-q_{Q}$ (the weights sum to one and equal 1/Q for equally spaced midpoint levels). This finite-support law ${\hat{F}}_{n}$ is a deliberate, low-complexity approximation rather than an exact reconstruction of the continuous predictive distribution. Algorithm 1 then implements (10) for that law. The role of the predictive law is easiest to see from the shape of D(k,·): over the SNR range in which rate decisions are made, the distortion surface is predominantly convex and decreasing in γ (Figure 2), because once the channel is good the residual distortion is dominated by the source bottleneck and saturates. By Jensen’s inequality, wherever the surface is convex over the support of ${\hat{F}}_{n}$,

(14) $$E_{\gamma ∼{\hat{F}}_{n}}\;\left[D(k,\gamma )\right]\;\geq \;D\left(k,E_{{\hat{F}}_{n}}\left[\gamma \right]\right),$$

so a plug-in controller underestimates expected distortion whenever the predictive law has non-zero spread and the surface is locally strictly convex. Near a rate boundary, that optimism can produce a rate that is too low. The effect is a consequence of curvature and is not removed merely by making the point forecast unbiased. Proposition 1 quantifies its leading-order contribution. The belief-averaged rule (10) itself does not rely on convexity: it evaluates the expectation directly, and convexity only determines the sign of the error that the plug-in rule makes.
**Algorithm 1** Prediction-aided risk-aware rate control, executed once per block *n* at the transmitter.**Require:** CSI window $O_{n}=\left\{{\hat{h}}_{n-\tau },\ldots ,{\hat{h}}_{n-\tau -W+1}\right\}$; fidelity target $D_{t}$; rate set K; distortion table D(·,·); quantile levels ${\left\{q_{i}\right\}}_{i=1}^{Q}$ and weights $\left\{w_{i}\right\}$**Ensure:** rate $k_{n}\in K$ and transmit vector $s_{n}$  1:$\left({\hat{\gamma }}_{n},\;{\left\{{\hat{\gamma }}_{n}^{\left(q_{i}\right)}\right\}}_{i=1}^{Q}\right)\leftarrow {\mathrm{LSTM}}_{\psi }\left(O_{n}\right)$     ▹ point + quantile heads  2:$k_{n}\leftarrow \max K$                                                           ▹ fallback  3:**for **k∈K in increasing order **do**  4:      ${\bar{D}}_{k}\leftarrow \sum_{i=1}^{Q}w_{i}\;D\left(k,{\hat{\gamma }}_{n}^{\left(q_{i}\right)}\right)$                               ▹ cf. (10)  5:      **if** ${\bar{D}}_{k}\leq D_{t}$ **then**  6:          $k_{n}\leftarrow k$; **break**                                                  ▹ smallest feasible  7:      **end if**  8:**end for**  9:$z_{n}\leftarrow f_{\theta }\left(x_{n},k_{n},{\hat{\gamma }}_{n}^{\left(0.5\right)}\right)$                                     ▹ forecast-conditioned10:$s_{n}\leftarrow \sqrt{2k_{n}}\;\left(m_{k_{n}}\;⊙z_{n}\right)/∥m_{k_{n}}\;⊙z_{n}∥$                                   ▹ cf. (2)

### 4.4. Construction of the Distortion Lookup Table

The table D(k,γ) is measured once after the codec has been trained and is never updated with test data. All 2317blocks of the validation split of the sensor streams are used for it. The SNR axis covers −8 to 30 dB in steps of 1 dB (39 points) and the rate axis covers the eight admissible rates, so the table has 312 entries. For each entry the encoder and decoder are conditioned on the grid SNR, the latent is truncated to *k* channel uses and passed through an AWGN channel at that SNR, and the energy-weighted aggregate NMSE (5) is computed over all validation blocks. The measurement is repeated for 4 independent noise draws, which are shared across entries so that neighboring entries are compared under identical noise, and the draws are averaged. The largest standard error of an entry is 0.0015, far below the distortion gap between adjacent rates.

Finite-sample fluctuations could make a larger rate appear marginally worse than a smaller one. The table is therefore projected to be non-increasing in *k* by the backward recursion $D\left(k_{i},\gamma \right)\leftarrow \max\left\{D\left(k_{i},\gamma \right),D\left(k_{i+1},\gamma \right)\right\}$; for the trained codec this projection changed 10 of the 312 entries. Between grid points the controller uses piecewise-linear interpolation in γ (in dB), and SNR values outside the grid are clamped to its end points. Linear interpolation preserves the monotonicity of the table in both arguments, and its convexity wherever the grid values are convex. Evaluating Algorithm 1 therefore requires at most |K|×Q=72 interpolations per block. Because the table is measured on the validation period of the streams, its values need not coincide with the distortion reached on the test period; for the NAB streams the validation period is harder to compress, so the table is conservative. All schemes share the same table, so this affects the operating targets but not the comparison between schemes.

### 4.5. Training Procedure

The semantic codec and the predictor are trained separately, which keeps the predictor interchangeable. The codec is trained for 110 epochs with Adam, an initial learning rate of $2\times 10^{-3}$ and a cosine schedule, on clean blocks of the training split with the rate *k* drawn uniformly from K per sample and the SNR drawn uniformly from [−5,25] dB, so that a single model spans the whole operating region; the nested latent is enforced by the random truncation. Drawing *k* separately for each sample rather than once per batch was necessary: with a per-batch rate the shared decoder failed to specialize and the rate ladder collapsed. The predictor is trained for 12 epochs with Adam (learning rate $3\times 10^{-3}$, cosine schedule, batch size 512) on simulated CSI trajectories at the deployment $f_{D}T$ and τ; the epoch with the lowest loss on the validation realizations is retained, and the calibration realizations are then used for the coverage check and for the optional offsets of Section 4.2. A single mobility-agnostic predictor, trained once on trajectories whose normalized Doppler is drawn log-uniformly from [0.0015,0.07], is also evaluated in Section 7.7, together with the fine-tuning of a trained predictor on a small adaptation set from a new regime (ten epochs, learning rate $10^{-3}$). At test time the decoder is conditioned on the true receive SNR, which the receiver estimates from the pilots, while the encoder is conditioned on the predicted SNR, so a prediction error affects both the rate choice and the encoder adaptation, exactly as it would in a deployed system.

## 5. Performance Analysis

We now relate the accuracy of the predictor to the distortion of the semantic link. Throughout, γ denotes the receive SNR in dB and $D^{\prime \prime }\left(k,\gamma \right)=\partial^{2}D\left(k,\gamma \right)/\partial \gamma^{2}$.

The analysis rests on two assumptions about the distortion response of the codec, stated separately because they play different roles.

**Assumption** **1.** *Forevery rate k and every operating point $\hat{\gamma }$ of interest, D(k,·) coincides, on a neighborhood of $\hat{\gamma }$ that carries all but a negligible part of the predictive law, with a three-time continuously differentiable function whose third derivative is bounded by a constant $M_{3}$.*

**Assumption** **2.** *On the same neighborhood, $D^{\prime \prime }\left(k,\gamma \right)\geq 0$.*

Assumption 1 is a statement about the smooth response of the trained codec, of which the lookup table of Section 4.4 is a sampled version; the proposition below is applied to that smooth response, and the piecewise-linear table is used only to evaluate it numerically. Assumption 1 alone gives the size of the plug-in error; Assumption 2 is needed only to fix its sign. Neither assumption is used by the controller, which evaluates (10) directly. Both are examined on the measured surface in Section 7.2.

**Proposition** **1.** *Let $\gamma =\hat{\gamma }+\varepsilon$ under the predictive law, where E[ε]=0, $E\left[\varepsilon^{2}\right]=\sigma_{e}^{2}$ and ${E[|\varepsilon |}^{3}]=O\left(\sigma_{e}^{3}\right)$. Under Assumption 1, for a fixed rate k,*

(15) $$E_{\gamma ∼\hat{F}}\left[D(k,\gamma )\right]=D\left(k,\hat{\gamma }\right)+\frac{1}{2}D^{\prime \prime }\left(k,\hat{\gamma }\right)\;\sigma_{e}^{2}+O\left(\sigma_{e}^{3}\right).$$

*Foroperating points away from a discrete rate boundary, so that the selected rate $k^{★}$ is locally unchanged by the forecast error, the expected bias of a plug-in distortion estimate is therefore*

(16) $$\Delta \;=\;\frac{1}{2}\;E\;\left[D^{\prime \prime }\left(k^{★},\hat{\gamma }\right)\;\sigma_{e}^{2}\right]\;+\;O\;\left(E[\sigma_{e}^{3}]\right),$$

*whereas the belief-averaged controller evaluates the expectation under the law supplied to it and therefore includes the second-order term. If, in addition, Assumption 2 holds, the leading term of* Δ *is non-negative.*

**Proof.**  Expand D(k,·) about $\hat{\gamma }$ to third order, $D\left(k,\gamma \right)=D\left(k,\hat{\gamma }\right)+D^{\prime }\left(k,\hat{\gamma }\right)\left(\gamma -\hat{\gamma }\right)+\frac{1}{2}D^{\prime \prime }\left(k,\hat{\gamma }\right){\left(\gamma -\hat{\gamma }\right)}^{2}+R_{3}$, and take the expectation under $\hat{F}$. The first-order term vanishes because $E_{\hat{F}}\left[\gamma \right]=\hat{\gamma }$, and the second-order term contributes $\frac{1}{2}D^{\prime \prime }\left(k,\hat{\gamma }\right)\sigma_{e}^{2}$. Taylor’s theorem and the bounded third derivative give $|R_{3}|\leq \frac{1}{6}M_{3}{\left|\gamma -\hat{\gamma }\right|}^{3}$ and hence $|E\left[R_{3}\right]|=O\left(\sigma_{e}^{3}\right)$, which proves (15). Applying the same expansion at locally stable decisions $k^{★}$ and averaging over operating points yields (16). Under Assumption 2, $D^{\prime \prime }\geq 0$ at every such point, so the leading term is non-negative. □

Two practical consequences follow. First, to leading order, the penalty of point-estimate control is proportional to the variance of the prediction error rather than to its standard deviation. This explains why forecast uncertainty weighs most on the rate decision in the high-mobility regime, where the error variance is largest. Second, the leading term depends on the predictor through its conditional variance, so a different probabilistic predictor can be substituted without changing the controller. Where the surface is locally concave, which for the trained codec happens only in a narrow low-SNR shoulder (Section 7.2), the same expansion shows that the plug-in rule errs on the pessimistic side instead; the belief rule remains correct in both cases.

Proposition 1 describes the distortion at a given rate. The controller, however, makes a discrete decision, and the blocks that matter most are those whose predicted distortion lies close to the target, where a small change of the estimate moves the decision to the next rate. The following result describes this switching behavior directly and shows that it obeys the same second-order law.

**Proposition** **2.** *Let $\hat{k}$ and $\tilde{k}$ denote the rates selected for a block by the plug-in rule and by the belief-averaged rule (10), with the notation of Proposition 1, and let $\hat{k}<\max K$. Under Assumptions 1 and 2, $\tilde{k}\geq \hat{k}$, and the two rules select different rates if and only if*

(17) $$D_{t}-\frac{1}{2}D^{\prime \prime }\left(\hat{k},\hat{\gamma }\right)\;\sigma_{e}^{2}+O\left(\sigma_{e}^{3}\right)<D\left(\hat{k},\hat{\gamma }\right)\leq D_{t}.$$

*Consequently, if p(·) denotes the density of the plug-in distortion estimate $D(\hat{k},\hat{\gamma })$ over the operating points, the probability that the two rules make different decisions is*

(18) $$\mathrm{Pr}\left\{\tilde{k}\neq \hat{k}\right\}=\frac{1}{2}\;p\left(D_{t}\right)\;E\left[D^{\prime \prime }\left(\hat{k},\hat{\gamma }\right)\;\sigma_{e}^{2}\;|\;D\left(\hat{k},\hat{\gamma }\right)=D_{t}\right]+O\left(\sigma_{e}^{3}\right),$$

*and, to the same order, a change of decision moves the rate by a single step of the rate ladder wherever the distortion gap between adjacent rates at $\hat{\gamma }$ exceeds $\frac{1}{2}D^{\prime \prime }\sigma_{e}^{2}$.*

**Proof.**  By Assumption 2 and Jensen’s inequality, $E_{\hat{F}}\left[D\left(k,\gamma \right)\right]\geq D\left(k,\hat{\gamma }\right)$ for every *k*, so every rate rejected by the plug-in rule is also rejected by the belief rule, and $\tilde{k}\geq \hat{k}$. The rate $\hat{k}$ is retained by the belief rule if and only if $E_{\hat{F}}\left[D\left(\hat{k},\gamma \right)\right]\leq D_{t}$; inserting (15) gives (17), while $D\left(\hat{k},\hat{\gamma }\right)\leq D_{t}$ holds by the definition of $\hat{k}$. Condition (17) places $D(\hat{k},\hat{\gamma })$ in an interval of width $\frac{1}{2}D^{\prime \prime }\left(\hat{k},\hat{\gamma }\right)\sigma_{e}^{2}+O\left(\sigma_{e}^{3}\right)$ just below the target, which yields (18). A move by two or more steps would additionally require the next rate to violate the target in expectation, i.e., its distortion at $\hat{\gamma }$ to lie in the same interval below $D_{t}$; since this distortion is smaller than $D(\hat{k},\hat{\gamma })$ by the gap between adjacent rates, this is excluded to second order whenever that gap exceeds $\frac{1}{2}D^{\prime \prime }\sigma_{e}^{2}$. □

Proposition 2 makes the role of the discrete decision explicit. Near a rate boundary the plug-in rule and the belief rule differ only in a band of width proportional to $D^{\prime \prime }\sigma_{e}^{2}$ below the target. Within this band the belief rule moves the block one step up the rate ladder, and the resulting reduction of the expected distortion is, to leading order, the gap between the two adjacent rates at $\hat{\gamma }$. Both the bias at a fixed rate and the frequency of a different decision therefore grow with the error variance, and both are numerically checked in Section 7.10.

The expansion also provides a practical criterion for when prediction is worth its complexity.

**Corollary** **1.** *Let $\sigma_{e,\mathrm{pred}}^{2}$ and $\sigma_{e,\mathrm{out}}^{2}$ denote the SNR-domain error variances of a predictor and of the outdated-CSI surrogate, respectively. At a common fixed rate and away from a rate boundary, and under Assumptions 1 and 2, replacing the surrogate by the predictor reduces the leading-order excess distortion whenever*

(19) $$E\;\left[D^{\prime \prime }\left(k,\hat{\gamma }\right)\sigma_{e,\mathrm{pred}}^{2}\right]<E\;\left[D^{\prime \prime }\left(k,\hat{\gamma }\right)\sigma_{e,\mathrm{out}}^{2}\right].$$

*If the curvature is sampled over the same operating region, the simpler condition $\sigma_{e,\mathrm{pred}}^{2}<\sigma_{e,\mathrm{out}}^{2}$ is sufficient.*

Equation (9) is stated for the complex channel gain rather than for SNR in dB, so the two error variances cannot be equated directly. It nevertheless shows the relevant trend: the aging component $2(1-J_{0}\left(2\pi f_{D}T\tau \right))$ vanishes as $f_{D}T\tau \to 0$, while the CSI estimation floor $\sigma_{\mathrm{csi}}^{2}$ remains. A local transformation to the SNR domain preserves this trend away from arbitrarily deep fades. The mobility-related benefit of prediction should therefore shrink in the quasi-static regime, even though a predictor may still provide some denoising gain. Section 7 tests this implication.

## 6. Experimental Setup

### 6.1. Real Datasets

Two kinds of real data are used: measured sensor streams as the semantic source, and measured cellular SNR logs as the channel.

We use six real streams from the Numenta Anomaly Benchmark (NAB) [23,24]: an industrial machine temperature sensor, an ambient temperature sensor, a server CPU utilization trace, a request-latency trace, the New York City taxi demand series, and a keystroke-timing trace. Using real streams rather than a synthetic generator matters here, because the achievable compression of a sensor block is a property of its actual spectral content. Each stream is standardized with statistics computed on its own training segment, split chronologically into 60% training, 15% validation and 25% test, and windowed into blocks of L=64 samples (stride 4 in the training and validation segments and 8 in the test segment). This yields 9545 training, 2317 validation and 1964 test blocks. The benchmark’s labeled anomaly windows are retained to define a downstream detection task.

The channel study of Section 7.8 uses the 5G production dataset of Raca et al. [25], recorded in the network of a major Irish mobile operator with the G-NetTrack Pro client on Android handsets. The logs contain client-side key performance indicators sampled about once per second, including the SNR reported by the handset, for two mobility patterns (static and driving) and several application patterns (video streaming and file download). From each log we keep the samples reported while the handset was attached to an LTE or 5G cell, average repeated entries within the same second, fill gaps of up to 3 s by linear interpolation, split the log at longer gaps and discard segments shorter than 120 s. The result is 59 driving logs (25.7 h) and 23 static logs (24.9 h) of per-second SNR. A single link-budget offset of 7.3 dB is added to all logs so that the mean SNR over the training logs is 10 dB, which places the traces in the same operating range as the fading study while leaving their measured temporal dynamics untouched. One semantic block is transmitted per second, so τ is expressed in seconds.

### 6.2. Separation of Training, Validation, Calibration and Test Data

Table 2 lists every dataset used in the study and its role. Three principles are applied throughout. (i) Sensor blocks are split chronologically, and the test blocks are used only for the final end-to-end evaluation. (ii) Simulated channel realizations for the training, validation, calibration and test roles are drawn from four independent random-number streams, generated from a common seed sequence with a distinct role key, so that no realization can appear in more than one role; the same holds for every mobility regime, delay and mismatch setting. (iii) Measured logs are assigned to roles as whole logs, interleaved in chronological order within each mobility class, so that the test logs are recordings that the predictor, the checkpoint selection and the calibration step have never seen. Within the test stage, all schemes are driven by common random numbers: the channel realization, the CSI noise and the additive noise are identical across schemes, so differences between schemes are paired.

### 6.3. Channel and CSI

Fading is generated by spectral shaping of complex white Gaussian noise with the Clarke spectrum of (6); the empirical autocorrelation of the generated process matches $J_{0}\left(2\pi f_{D}Tm\right)$ to three decimal places. We use this analytically tractable model rather than a tapped-delay-line profile such as 3GPP TDL-C [42] because the Wiener baseline requires a closed-form autocorrelation to be genie-aided at all; the mismatch studies below relax the model in two important directions, and the measured logs remove the model altogether. We sweep $f_{D}T\in \left\{0.002,0.005,0.01,0.02,0.05\right\}$ and τ∈{1,2,5,10} blocks, with an average SNR of ρ=10 dB and a CSI estimation SNR of 15 dB, i.e., $\sigma_{\mathrm{csi}}^{2}=10^{-1.5}$. The observation window is W=24 blocks. Two mismatched deployments are used for the robustness study, chosen to reflect two ways in which the assumptions of the model-based baseline can fail in a real installation. In the spectral-shape mismatchthe environment contains two scatterer clusters with maximum Doppler $f_{D}T$ and $0.35f_{D}T$ and weights 0.55/0.45, plus a Rician component with K=3 dB; the single-cluster Clarke shape assumed by the Wiener filter is then wrong, although its assumed time scale remains roughly right. In the Doppler mismatch the shape is Clarke but the maximum Doppler itself is unknown: each realization multiplies the nominal $f_{D}T$ by a factor drawn uniformly from {0.5,1,2,4}, modeling a node whose mobility is not known at design time. The Wiener filter is designed for the nominal $f_{D}T$ and is therefore mis-tuned by up to a factor of four, whereas the learned predictor is trained on the mixture without being told what varies and must infer the time scale from the observation window itself.

Three further settings bring the simulated link closer to practical deployments; they are evaluated in Section 7.9. (i) **Non-stationary Doppler**: Within every realization the maximum Doppler drifts smoothly between $f_{D}T=0.002$ and 0.05 over a few hundred blocks, as for a node that speeds up and slows down. The channel is a sum of 64 sinusoids whose phases accumulate the instantaneous Doppler, so the fading rate changes continuously along the record. (ii) **Time-varying CSI quality**: At $f_{D}T=0.02$, the CSI estimation SNR changes in piecewise-constant segments of random length (mean 200 blocks), each drawn uniformly between 5 and 25 dB, as when the pilot quality changes with interference or load. (iii) **Frequency-selective multipath**: A four-tap channel with an exponential power delay profile (relative powers 0.60, 0.26, 0.11 and 0.02 at delays of 0, 1, 2 and 4 samples), each tap fading independently with the Clarke spectrum, is observed through 64 orthogonal frequency-division multiplexing (OFDM) subcarriers at $f_{D}T=0.02$ and 0.05. The codec is driven by the capacity-equivalent effective SNR across the subcarriers, and the transmitter receives delayed reports of the same quantity computed from noisy subcarrier estimates at 15 dB. In settings (i) and (ii) no predictor is retrained: the Wiener filter keeps its nominal design ($f_{D}T=0.01$ and 0.02, respectively) and the learned predictors are those of the preceding experiments. In setting (iii) the transmitter sees only a scalar report, so a scalar-input LSTM is trained once over $f_{D}T\in \left[0.0015,\;0.07\right]$ and compared with a data-driven linear minimum mean-squared error (LMMSE) predictor fitted on the same training windows, since no closed-form autocorrelation of the effective SNR is available.

### 6.4. Schemes Compared

Seven schemes share the same codec, the same distortion table and the same noise realizations; they differ only in ${\hat{F}}_{n}$.

- **Fixed rate**: No adaptation; every block uses the admissible rate equal to the reference bandwidth.
- **Outdated CSI**: Plug-in control on ${\hat{h}}_{n-\tau }$, i.e., the idealized adaptive designs of [12,13] operated with realistic feedback.
- **Wiener (point)**: Genie-aided LMMSE prediction of $h_{n}$ from $O_{n}$, given the exact $f_{D}$ and $\sigma_{\mathrm{csi}}^{2}$, used as a point estimate with the conditional-mean correction $E[{\left|h_{n}\right|}^{2}\;|\;O_{n}]={\left|{\hat{h}}_{n}\right|}^{2}+\sigma_{e}^{2}$.
- **Wiener (belief)**: The same predictor, but with its exact conditional law $\mathrm{CN}({\hat{h}}_{n},\sigma_{e}^{2})$ propagated through (10) by Monte Carlo with 24 samples per block. Under a matched Clarke spectrum this is the Bayes-optimal predictive law and therefore the strongest possible reference.
- **LSTM (point)** and **LSTM (belief, proposed)**: The learned predictor of Section 4, used respectively through its point head and its quantile head. Comparing the two isolates the contribution of the predictive law.
- **Genuine CSI**: Unattainable reference with ${\hat{F}}_{n}=\delta_{\gamma_{n}}$.

For the measured logs the Wiener filter cannot be genie-aided, because the true autocorrelation is unknown. It is replaced by its data-driven counterpart: an LMMSE predictor with *W* taps and an intercept, fitted by least squares to the SNR sequences of the training logs, used either as a point forecast (LMMSE (point)) or with a Gaussian predictive law whose variance is the training residual variance (LMMSE (belief)).

### 6.5. Evaluation Protocol

For the same target $D_{t}$, different schemes spend different average bandwidths, so comparing them at a common $D_{t}$ would mix a bandwidth effect into the comparison. For every scheme we therefore tune $D_{t}$ by bisection until the average number of channel uses per block over the whole test set equals the reference value ${\bar{k}}_{\mathrm{ref}}$. Every block still uses an admissible rate from K; only the average is matched, and no interpolation between transmission rates is involved. Because the belief-averaged distortion changes continuously with the forecast, the matched average is reached exactly on the simulated channels and to within 0.036 channel uses per block on the measured logs, whose SNR values are reported with a resolution of 1 dB. The fixed-rate link uses $k={\bar{k}}_{\mathrm{ref}}$ for all blocks. The main reference is ${\bar{k}}_{\mathrm{ref}}=12$; trade-off curves repeat the matching for ${\bar{k}}_{\mathrm{ref}}\in \left\{4,6,\ldots ,14\right\}$, and selected results are also given for ${\bar{k}}_{\mathrm{ref}}=8$.

Every simulated operating point uses 20 independent test channel realizations, each carrying the 1964 test blocks, i.e., 39,280 block transmissions per scheme. Metrics are computed per realization, and we report the mean together with a 95% confidence interval from the Student-*t* distribution over realizations. Differences between the proposed scheme and a baseline are assessed on the paired per-realization differences with the Wilcoxon signed-rank test [43]. For the measured logs each test log is one realization; because the logs differ in length, means are weighted by the number of blocks and confidence intervals are obtained with a log-level bootstrap (5000 resamples).

All training, simulation and timing runs were executed on a Linux virtual machine with an Intel^®^ Xeon^®^ processor at 2.80 GHz (two logical cores allocated) and 7 GB of memory, using Python 3.11 and PyTorch 2.14.0 on the CPU only; no GPU was used at any stage.

## 7. Results and Discussion

### 7.1. Channel Prediction Accuracy and Calibration

Figure 3a shows a segment of the receive SNR process at $f_{D}T=0.02$ together with the outdated surrogate, the LSTM point forecast and the 90% predictive band. The outdated trace is visibly shifted in time, and it deviates most in the deep fades, which dominate the distortion. Figure 3b reports the prediction root-mean-squared error (RMSE) in the SNR domain over the Doppler sweep. The error of the outdated surrogate grows steeply with mobility, from 2.15 dB at $f_{D}T=0.002$ to 7.28 dB at $f_{D}T=0.05$, while the errors of both predictors grow far more slowly; at $f_{D}T=0.05$ the LSTM reaches 4.94 dB. The genie-aided Wiener predictor retains a small edge over the LSTM under a matched spectrum (4.30 dB at $f_{D}T=0.05$), which is expected since it is the Bayes-optimal linear estimator for exactly this process.

Calibration is reported in Figure 4 as a reliability diagram, and Table 3 gives the corresponding numerical measures on the test realizations: the average coverage error $\mathrm{ACE}=\frac{1}{Q}\sum_{i}\left|{\hat{c}}_{i}-q_{i}\right|$, where ${\hat{c}}_{i}$ is the empirical frequency with which the true SNR falls below the predicted $q_{i}$-quantile; the prediction interval coverage probability ${\mathrm{PICP}}_{90}$, that is, the empirical coverage of the central 90% interval between the 5% and 95% quantiles, together with its mean width; and the mean pinball loss, a proper scoring rule that rewards calibration and sharpness jointly [35]. Across the whole Doppler range the empirical coverage of the nine predicted quantiles follows the nominal levels closely: the ACE does not exceed 0.023. The central interval is slightly too narrow, with ${\mathrm{PICP}}_{90}$ between 0.852 and 0.885 against the nominal 0.90, because the outermost levels are learned from comparatively few tail samples. The width of the interval grows with mobility, from 2.4 dB at $f_{D}T=0.002$ to 15.1 dB at $f_{D}T=0.05$, so the predictive law widens where the forecast is harder, while its coverage stays close to the nominal level. The optional recalibration of Section 4.2 (column “ACE, recal.”) changes the ACE by at most 0.044 on simulated channels, in either direction. On the measured logs it is harmful: the calibration logs are other recordings, and the offsets estimated on them raise the ACE on the test logs from 0.036 to 0.078 in the driving case with τ=5 s. This is why the proposed scheme uses the quantile head without offsets. Under the Doppler mismatch, the exact conditional law of the mis-tuned Wiener filter has an ACE of 0.131 and a 90% coverage of only 0.523, whereas the LSTM belief keeps an ACE of 0.011 and a coverage of 0.861. This is the property that makes (10) meaningful: an over-dispersed forecast would meet the target by wasting bandwidth, and an under-dispersed one would reproduce the plug-in bias.

### 7.2. The Distortion Surface

Figure 2a shows the measured D(k,γ) of the trained codec. Two features of this surface shape the results that follow. The surface is monotone in *k*, so the rate ladder is usable; and it is convex in γ over the range in which rate decisions are made, steep at moderate SNR and flat at high SNR, so an optimistic SNR error costs much more than a pessimistic error of the same magnitude. This is the asymmetry that Proposition 1 formalizes.

Figure 2b checks Assumptions 1 and 2 directly. It shows the second difference of the table on the 1 dB grid, with a band of two standard errors. The standard errors are estimated from the second differences of the individual noise draws, so they account for the noise shared between neighboring entries. The second difference varies smoothly with γ, as Assumption 1 requires. It is positive with statistical significance from 2 dB upward for every rate, until the surface becomes flat above about 20 dB, where its magnitude stays below $10^{-3}$. Significantly negative values appear only in a narrow shoulder between −3 and 0 dB, where the reconstruction starts to saturate toward the source variance. This shoulder has little influence on the controller: at $\bar{k}=12$ the predicted quantiles of the blocks whose decision lies close to a rate boundary place only 6.9% ($f_{D}T=0.02$) and 7.3% ($f_{D}T=0.05$) of their probability mass in it. Assumption 2 therefore holds for all but a small part of the decisions, and where it fails the plug-in error changes sign, while the belief rule is unaffected.

### 7.3. Bandwidth–Distortion Trade-Off

Figure 5 shows the trade-off between average bandwidth and achieved semantic distortion at two mobility levels, with every point obtained by matching all schemes to the same average bandwidth. At $f_{D}T=0.01$ and for $\bar{k}\geq 10$ the ordering is the expected one: the fixed-rate link is worst, outdated-CSI adaptation improves on it, prediction improves further, and the genuine-CSI bound is best. At $f_{D}T=0.05$ the picture changes qualitatively: the outdated-CSI curve rises above the fixed-rate curve over the whole bandwidth range, so the adaptation mechanism is no longer merely less useful but counterproductive. The vertical spacing between the outdated and the genuine-CSI curves is the quantity introduced in Section 1 as the semantic adaptation mismatch, and it widens with mobility. At small average bandwidths ($\bar{k}\leq 8$) the fixed-rate link is competitive with every adaptive scheme, including the genuine-CSI one. This is a property of the fidelity-target rule (10) rather than of the CSI: a loose target lets the rule send well-received blocks at the smallest rates, whose distortion is limited by the source bottleneck, while the few faded blocks receive the largest rate. Such low-fidelity operating points are outside the intended use of a fidelity-target controller, and the remaining comparisons concentrate on $\bar{k}=12$, where the tuned targets lie around $D_{t}\approx 0.27$.

The open markers in Figure 5 are the operating points that each scheme reaches directly for seven fidelity targets between $D_{t}=0.14$ and $D_{t}=0.40$, without any matching or interpolation; for the fixed-rate link they are its admissible rates. The matched curves run through these raw points for every scheme. Reading a common bandwidth off the raw points by linear interpolation, as is often done, would shift the distortion at $\bar{k}=12$ by at most 0.0010 and the outage by at most 0.25 percentage points (for $f_{D}T\in \left\{0.01,0.02,0.05\right\}$). Because the operating curves are convex, the interpolated values lie slightly above the exact ones. Such shifts can reverse the order of two schemes whose results differ by less than this amount, but they leave every conclusion of this section and of Section 7.4 unchanged, and the exact matching used throughout the paper avoids them altogether.

### 7.4. Effect of Mobility and Feedback Delay

Figure 6a reports the distortion of every scheme at a common average bandwidth of $\bar{k}=12$ channel uses per block as mobility increases, and Figure 6b expresses the same data as the fraction of the outdated-to-genuine gap that each predictor closes. Error bars are 95% confidence intervals over the 20 test realizations, and Table 4 lists the values at $f_{D}T=0.02$ and 0.05 together with the outcome of the paired tests.

The most important feature of Figure 6a is the crossing of the outdated-CSI and fixed-rate curves. At low mobility the ordering is the expected one and the outdated-CSI scheme sits comfortably below the fixed-rate curve, since even stale CSI carries useful information. At $f_{D}T=0.02$ the two curves meet ($\bar{D}=0.1684$ against 0.1686), and in outage the outdated-CSI scheme is already the worse of the two (21.0% against 20.5%). At $f_{D}T=0.05$ the outdated-CSI scheme has become the worst of the seven: $\bar{D}=0.1860$ against 0.1723 for a fixed-rate link of the same average bandwidth, and a semantic outage of 23.5±0.5% against 20.9±0.6% (paired test, $p<10^{-3}$). The same inversion appears in the delay sweep of Figure 7: at τ=10 blocks and $f_{D}T=0.01$, outdated-CSI adaptation reaches an outage of 21.2% against 20.7% for the fixed-rate link. The interpretation is that once the CSI is old enough, the controller’s decisions become negatively correlated with the channel it actually meets: it raises the rate when the channel has already recovered and lowers it when the channel is about to fade. An adaptive semantic scheme evaluated only under instantaneous CSI would not reveal this behavior. The inversion does not depend on the particular requirement used for outage: at $f_{D}T=0.05$, with $D_{\mathrm{req}}=0.20$ the outdated-CSI and fixed-rate links reach 28.2% and 25.3%, and with $D_{\mathrm{req}}=0.30$ they reach 19.8% and 17.7%.

With prediction, this loss relative to the fixed-rate link disappears. At $f_{D}T=0.05$ the proposed scheme attains $\bar{D}=0.1723$ against the bound 0.1567, closing 47% of the penalty, and the genie-aided Wiener scheme closes 52%. The proposed scheme thereby brings the link back to the performance of the fixed-rate link ($\bar{D}=0.1723$), whereas at $f_{D}T=0.02$ and below it improves significantly on both the fixed-rate link and outdated-CSI adaptation (Table 4). The behavior at low mobility follows Corollary 1: at $f_{D}T=0.002$ the channel barely moves over the feedback delay, all adaptive schemes converge to within 0.004 in distortion, and prediction provides almost no mobility-related advantage.

Figure 7 repeats the comparison in terms of semantic outage probability. Outage is especially sensitive to rate under-provisioning and separates the schemes more clearly than mean distortion, which is diluted by the many blocks transmitted under comfortable channel conditions.

Comparing LSTM (point) with LSTM (belief) isolates the effect of the predictive law. Once both are tuned to the same average bandwidth, their average differences are small: over all Clarke configurations and both reference bandwidths the outage values differ by at most 0.7 percentage points. The belief is ahead at $\bar{k}=8$, where more decisions fall on the steep part of the surface (outage 29.9% against 30.3% at $f_{D}T=0.01$, 28.4% against 29.1% at 0.02 and 27.5% against 27.9% under the Doppler mismatch), while at $\bar{k}=12$ and $f_{D}T=0.05$ the point head is marginally ahead. The reason is that tuning $D_{t}$ to a common bandwidth already absorbs the average optimism of the plug-in rule; what remains is the block-to-block variation of the forecast uncertainty, which the belief takes into account and the point forecast does not. The belief therefore does not bring a large average gain at a common bandwidth. Its role is to make the expected distortion that the controller acts on consistent with the forecast uncertainty (Section 7.10), and to keep the decisions reliable when the predictor’s uncertainty is well calibrated but the model behind a competing predictor is wrong (Section 7.6).

### 7.5. Results for Individual Sensor Streams

Table 5 breaks the results at $f_{D}T=0.05$ and $\bar{k}=12$ down by sensor stream. The streams differ strongly in how well the codec can represent them. The two temperature streams and the taxi demand series are smooth and are reconstructed with distortions between 0.06 and 0.12; the CPU utilization trace lies between these and the two impulsive traces; and the request-latency and keystroke traces, which consist of isolated spikes on a flat background, remain above 0.4 even with genuine CSI, so their outage is dominated by the source rather than by the channel. The relative behavior of the schemes is consistent across streams: outdated-CSI adaptation has the highest distortion in every stream, prediction removes most of that loss, and the genuine-CSI bound has the lowest distortion in every stream except the request-latency trace, where all schemes are within the confidence intervals. For the two impulsive streams the channel-related differences are small compared with the source-related distortion. Because a single distortion table serves all streams, the controller does not exploit these differences; a stream-specific table is a direct extension that requires no change to the predictor.

### 7.6. Robustness to Doppler-Spectrum Mismatch

The genie-aided Wiener predictor is the strongest baseline because it is handed the true second-order statistics, and under the matched Clarke process this advantage shows: at $f_{D}T=0.02$ its SNR prediction RMSE is 2.50 dB against 3.18 dB for the LSTM, and it closes a correspondingly larger share of the outdated-CSI penalty (60% against 44%). This is expected, because a linear estimator that knows the exact statistics of a Gaussian process is optimal for that process. The question this subsection asks is what happens when that knowledge is not available, which is the usual situation in a real deployment.

The spectral-shape mismatch has only a mild effect. At $f_{D}T=0.02$ the prediction RMSE becomes 1.92 dB for the Wiener filter and 2.06 dB for the LSTM, and end-to-end the two remain close, recovering 66% and 54% of the outdated-CSI penalty respectively. Getting the shape of the Doppler spectrum wrong while its time scale stays right costs the Wiener filter little: the autocorrelation it assumes is still approximately correct over the few blocks that matter for a τ=5 horizon.

Under the Doppler mismatch the picture changes, as Figure 8 shows. When the maximum Doppler is unknown to within a factor of four, the Wiener filter’s prediction RMSE degrades to 6.42 dB, worse than both the LSTM (4.36 dB) and the outdated CSI itself (5.89 dB): the ordering established above is reversed, and by a wide margin. The learned predictor can infer the prevailing time scale from the observation window, whereas the linear filter has that time scale fixed in its coefficients and cannot. The end-to-end consequence is clear. At a nominal $f_{D}T=0.02$ the model-based scheme recovers only 14% of the outdated-CSI penalty against 39% for the proposed scheme, and its distortion ($\bar{D}=0.1743$) is higher than that of the fixed-rate link (0.1725), while the proposed scheme reaches $\bar{D}=0.1690$ and an outage of 20.8% against 22.4% for outdated-CSI adaptation. Because the Doppler factor differs from one realization to the next, the per-realization results of the mis-tuned filter vary widely, which is visible in the wider confidence intervals of Table 4. At the lower nominal Doppler of 0.005 the same pattern appears in milder form: the Wiener scheme recovers 33% of the penalty, against 37% for the proposed one.

We draw a narrower conclusion from this result than learning beats model-based estimation. A mis-tuned Wiener filter reports an error variance $\sigma_{e}^{2}$ computed from its assumed statistics, which is far smaller than the error it actually makes. The belief it passes to the controller is therefore over-confident (its central 90% interval covers the true SNR only 0.523 of the time, Table 3) and, by Proposition 1, the controller under-provisions. The learned predictor’s quantile head is calibrated against the deployment distribution rather than against an assumed model, so its predictive interval widens as conditions worsen (coverage 0.861). What this experiment isolates is therefore not raw estimation accuracy but the trustworthiness of the uncertainty, which is the quantity a risk-aware controller consumes.

### 7.7. Generalization and Adaptation Across Mobility

In the preceding experiments a predictor was trained for each mobility regime. Training a new model for every condition a node may meet is rarely practical, so this subsection examines two ways around it. Figure 9 compares four options on the same test realizations: the regime-specific predictors; a single mobility-agnostic predictor trained once over $f_{D}T\in \left[0.0015,\;0.07\right]$ (Section 4); the predictor trained only at $f_{D}T=0.01$ and deployed unchanged at every other Doppler; and the same predictor after a short fine-tuning on a small amount of data from the new regime.

The single-regime predictor, deployed without adaptation, behaves as expected from a model applied outside its training conditions. At $f_{D}T=0.05$ its RMSE of 7.92 dB exceeds that of the outdated CSI, and its outage of 24.0% is worse than outdated-CSI adaptation (23.5%), so a predictor tuned for the wrong mobility can be worse than no prediction at all. The mobility-agnostic predictor avoids this without being told the Doppler: its RMSE stays within 0.43 dB of the regime-specific models at every tested mobility (5.30 dB against 4.94 dB at $f_{D}T=0.05$), it closes 38% and 42% of the outdated-CSI penalty at $f_{D}T=0.02$ and 0.05 (regime-specific: 44% and 47%), and its outage at $f_{D}T=0.05$ is 21.2% against 20.9%. Under the Doppler mismatch, where the node’s mobility changes from one realization to the next, the same single model reaches an outage of 21.0% at nominal $f_{D}T=0.02$, close to the 20.8% of the predictor trained on that mixture. A single predictor can therefore serve a node whose mobility is unknown or variable, at a small cost relative to regime-specific training.

When a node enters a regime that its predictor was not trained for, the existing model can be adapted rather than replaced. Starting from the predictor trained at $f_{D}T=0.01$, we used 3484 training windows from the new regime (four sequences of 900 blocks, one sixth of the data used for regime-specific training) and compared two forms of adaptation: retraining only the two output heads with the LSTM layers frozen, and fine-tuning all weights for ten epochs at a learning rate of $10^{-3}$. At $f_{D}T=0.05$, retraining the heads lowers the RMSE from 7.92 dB (no adaptation) to 7.06 dB, and full fine-tuning lowers it to 5.46 dB, close to the 4.94 dB of the regime-specific model. The end-to-end result follows the same order: the fine-tuned predictor closes 44% of the outdated-CSI penalty with an outage of 21.1%, against 47% and 20.9% for the regime-specific model, while retraining the heads alone closes 21%. At $f_{D}T=0.02$ fine-tuning closes 43% with an outage of 19.9% (regime-specific: 44% and 19.8%), and it also carries the model toward lower mobility, where the RMSE at $f_{D}T=0.002$ drops from 1.85 to 1.35 dB. Because retraining the heads recovers only part of the benefit, the recurrent layers themselves must also adjust to the new time scale of the fading; this adjustment needs only a few epochs, which makes full fine-tuning the more useful option.

In terms of training cost, on the hardware described in Section 6.5, training a regime-specific predictor from scratch takes about 55 s (20,904 windows, 12 epochs), and the mobility-agnostic predictor, which sees more varied data, takes about 93 s once. Fine-tuning takes at most 6 s and retraining the heads slightly less. None of these options changes the inference cost of Table 10, since the architecture is the same, and none requires the codec or the distortion table to be touched. In practice, a node can therefore start from the mobility-agnostic predictor, and when its operating conditions settle for long enough to collect a few thousand reports, it can refine the model locally within seconds or upload the reports for fine-tuning at an edge server.

### 7.8. Evaluation on Measured 5G/LTE Channel Logs

The fading model gives control over mobility and delay, but it also fixes the statistics of the channel. The measured logs of Section 6 remove that assumption: the SNR sequences come from handsets moving through, or resting in, a commercial network, and they contain slow trends from path loss and shadowing together with abrupt steps caused by cell changes, switching between LTE and 5G carriers and blockage. To our knowledge, this is the first time that semantic rate control under outdated CSI has been evaluated on such recordings. Figure 10a shows an excerpt of a test drive with a feedback delay of 5 s, and Table 6 and Figure 10b summarize the end-to-end results on the 11 driving and 4 static test logs at $\bar{k}=12$.

Three observations stand out. First, the failure mode of Section 7.4 is present in real data. In the driving logs, outdated-CSI adaptation lowers the distortion from 0.1414 (fixed-rate link) to 0.1300 when the feedback is 1 s old, but only from 0.1423 to 0.1386 when it is 5 s old, and its outage advantage has then disappeared (16.8% against 16.9%). In the static logs with τ=5 s it is worse than the fixed-rate link in both distortion (0.1380 against 0.1365) and outage (16.8% against 16.5%), although with only four static test logs this difference is not statistically significant.

Second, prediction restores the benefit of adaptation. For τ=5 s in the driving logs the LSTM reduces the SNR prediction error from 7.40 dB to 6.34 dB and the proposed scheme reaches $\bar{D}=0.1359$ and an outage of 16.2%, both significantly better than outdated-CSI adaptation; in the static logs it reaches 0.1356 against 0.1380. The share of the outdated-CSI penalty that is recovered, 21% for driving at τ=5 s, is lower than in simulation. Much of the second-scale variation of a measured SNR log cannot be anticipated from its past, which leaves a clear gap to the genuine-CSI bound for every causal scheme.

Third, the data-driven linear predictor performs as well as the LSTM on these logs, in contrast to the Doppler-mismatch study. The predictable part of a measured per-second SNR log is dominated by slow trends that a linear filter captures well, while the learned model’s advantage in simulation came from inferring an unknown Doppler time scale. The quantile head of the LSTM keeps a moderate coverage error of 0.036 on the driving test logs (Table 3), so the belief transfers to recordings the predictor has never seen. The results on measured data thus confirm the two central findings of the simulation study (the loss caused by outdated CSI and its recovery by prediction) in a setting where no channel model is assumed at all.

### 7.9. Non-Stationary, Noisy and Frequency-Selective Channels

The flat and stationary Clarke channel keeps the model-based baseline well defined, but a deployed link usually departs from it in more than one way. Table 7 reports the three additional conditions described in Section 6, evaluated exactly as before: at $\bar{k}=12$ and τ=5, with every scheme tuned to the same average bandwidth, with the same outage requirement and over 20 test realizations.

Under non-stationary Doppler, the mobility drifts within a realization, so no single Doppler design fits the whole record. The Wiener filter designed for $f_{D}T=0.01$ then predicts less accurately than the outdated report itself (RMSE 5.03 against 4.50 dB) and gives no end-to-end gain over it. The mobility-agnostic predictor of Section 7.7, applied without any retraining, follows the drift from its observation window: its RMSE is 3.40 dB, it closes 38% of the outdated-CSI penalty, and it lowers the outage from 21.0% to 20.0%.

When the estimation SNR moves between 5 and 25 dB, the delayed report is at times dominated by estimation noise, and outdated-CSI adaptation becomes worse than the fixed-rate link ($\bar{D}=0.1700$ against 0.1676). The Wiener filter keeps the correct Doppler in this setting and averages over the *W* past reports, which suppresses part of the extra noise, so it remains the most accurate predictor (RMSE 2.83 dB) and closes 55% of the penalty. The learned predictor, trained at a fixed CSI SNR of 15 dB and not retrained, still closes 43% and lowers the outage from 21.3% to 20.0%, close to the 19.9% of the Wiener filter. With the same learned predictor, a decision on the point forecast reaches the same average distortion but a higher outage (20.3%), so the belief-based rule is again the more reliable of the two.

On the frequency-selective channel, averaging over 64 subcarriers removes much of the deep fading of a flat channel: the standard deviation of the effective SNR is about 2.9 dB, and even genuine CSI improves on the fixed-rate link by less than 0.004 in $\bar{D}$. Within this narrower range the ordering seen on flat channels is preserved. At $f_{D}T=0.02$ the scalar-input LSTM is the most accurate predictor (RMSE 1.29 dB, against 1.59 dB for the LMMSE predictor and 1.86 dB for the outdated report) and closes 48% of the outdated-CSI penalty. At $f_{D}T=0.05$ outdated-CSI adaptation is again worse than the fixed-rate link, as on the flat channel at the same mobility. The LSTM again predicts best (RMSE 2.74 dB) and brings the adaptive link back to within 0.001 of the fixed-rate distortion with the same outage, whereas the LMMSE predictor stays further away. Frequency diversity thus reduces how much any adaptive scheme can gain, but wherever adaptation is used, prediction is what prevents aged reports from turning it into a loss.

### 7.10. Numerical Illustration of the Second-Order Analysis

Figure 11 illustrates Propositions 1 and 2 on the measured distortion surface, independently of the fading-channel experiments. Operating points $\hat{\gamma }$ are drawn from the SNR distribution of a Rayleigh channel at 10 dB, the plug-in rule selects $\hat{k}$ for a target of 0.24, and the true SNR is $\hat{\gamma }+\varepsilon$ with a synthetic error of controlled standard deviation $\sigma_{e}$; the belief rule receives the corresponding Gaussian law. All quantities are averaged over the operating points with $\hat{\gamma }\geq 0$ dB (91% of all points), where Assumption 1 holds (Section 7.2).

Figure 11a compares the measured bias of the plug-in estimate, $E_{\varepsilon }\left[D\left(\hat{k},\hat{\gamma }+\varepsilon \right)\right]-D\left(\hat{k},\hat{\gamma }\right)$, with the second-order term $\frac{1}{2}D^{\prime \prime }\left(\hat{k},\hat{\gamma }\right)\sigma_{e}^{2}$ of (16). The two agree closely up to $\sigma_{e}\approx 2$ dB, remain within about 10% of each other at 3 dB, and diverge only when the expansion is no longer justified, which is the expected behavior of a second-order result. Below 0 dB the surface bends sharply as the reconstruction saturates, and the leading term no longer describes the bias there.

Figure 11b turns to the discrete decision. It shows the fraction of operating points at which the belief rule selects a different rate from the plug-in rule, together with the fraction predicted by condition (17) of Proposition 2. The prediction follows the measured switching closely over the whole range: at $\sigma_{e}=2$ dB the two rules disagree on 6.1% of the operating points against 6.1% predicted, and at $\sigma_{e}=3$ dB on 13.4% against 14.0%. In agreement with the proposition, every change of decision observed is a single step of the rate ladder, and the reduction of the expected distortion that it brings, 0.0061 at $\sigma_{e}=3$ dB, is close to the value 0.0067 obtained from the gap between adjacent rates. Figure 11c shows the mechanism in terms of resources: the belief-averaged controller spends slightly more bandwidth as uncertainty grows, thereby reducing the optimistic under-provisioning produced by plug-in control.

### 7.11. Sensitivity to the Quantile Representation and the Decision Criterion

Table 8 varies the two design choices that the controller depends on. For each quantile set a separate predictor was trained with the same protocol, and every variant is again matched to a common bandwidth.

At $\bar{k}=12$ the results are practically insensitive to the number and placement of the quantile levels: for all sets from a single median up to 19 equal-mass levels, the distortion stays within 0.001 and the outage within about 0.1 percentage points of the default. At $\bar{k}=8$, where more decisions fall on the steep part of the surface, the differences grow to about 0.002 in distortion. Two trends are visible there. Very fine sets do not help, because the outermost levels of a 19-level set (q=0.026 and 0.974) are learned from few tail samples and require the largest recalibration offsets. Placing more levels in the lower tail, where the SNR is low and the surface is steep, gives the lowest distortion at $f_{D}T=0.05$. Nine levels are therefore a sound default: they are accurate enough in the tails that matter, cheap to evaluate, and include the 90% interval used for calibration monitoring. A lower-tail-dense placement is a small refinement for strongly time-varying links.

The same table compares the risk-aware rule (10) with its point-forecast counterpart, with the recalibrated quantiles, and with the quantile rule (11) that directly constrains the predictive outage probability. The optional recalibration changes the outage by less than 0.1 percentage points on simulated channels. The quantile rule is clearly inferior at a common bandwidth, particularly for ε=0.05: protecting the lower tail of every block spends bandwidth on blocks whose channel is likely to be poor anyway, and that bandwidth is then missing from the blocks that could have been improved. Constraining the expected distortion is thus the better use of the predictive law when bandwidth is the scarce resource.

### 7.12. Downstream Task and Complexity

Table 9 reports three task-level measures at $f_{D}T=0.05$, τ=5. The first is the anomaly-detection $F_{1}$ score of a detector trained on clean validation blocks and applied to the reconstructed test blocks. Its values lie between 0.473 and 0.476 for all schemes, against 0.489 on the clean source, and the differences between schemes, at most 0.003, lie within or close to the confidence intervals and are too small to rank the schemes. We therefore do not draw conclusions about semantic improvement from this metric. It shows only that the detector, which relies on coarse block-level descriptors, keeps its decisions under all schemes. The second measure makes this explicit: the fraction of blocks on which the decision made on the reconstruction agrees with the decision made on the clean block lies between 0.907 and 0.915.

Because a detector of this kind is insensitive to small reconstruction errors by design, we added a task whose decisions depend on the reconstructed values themselves: a level-crossing alarm that flags every sample exceeding a critical level, set for each stream at the 95th percentile of its training segment, as in threshold-based condition monitoring. The alarm is evaluated with the $F_{1}$ score against the alarms raised on the clean stream. This task clearly separates outdated-CSI adaptation from the other schemes. At $\bar{k}=12$, outdated-CSI adaptation reaches 0.454, the fixed-rate link 0.487, the proposed scheme 0.494 and the genuine-CSI bound 0.508. At $\bar{k}=8$ the gap widens: outdated-CSI adaptation falls to 0.354, while the proposed scheme reaches 0.437. The clear effect is therefore the loss caused by outdated CSI and its removal by prediction, in line with the outage results. The differences among the prediction-based schemes, the fixed-rate link and the genuine-CSI bound are much smaller, at most about 0.02 in $F_{1}$ at $\bar{k}=12$ and largely within the confidence intervals, so we do not use them to rank these schemes. Moreover, at $\bar{k}=8$ the genuine-CSI bound does not reach the highest value, which reflects the behavior of the fidelity-target rule at small bandwidths discussed in Section 7.3 rather than a property of the CSI.

Table 10 reports the computational cost of the added components. All latencies were measured on the platform described in Section 6.5, for a single block (batch size one) in float32, after 50 warm-up runs, and are given as the median together with the 10th and 90th percentiles over 2000 to 5000 runs; the single-thread setting is the closer proxy for an embedded gateway processor. The predictor is a two-layer LSTM with 64 hidden units, 52 kparameters and about 1.2 million multiply-accumulate operations per block, a count that does not depend on the platform. Its inference cost of 0.60 msper block is far below the block duration in any realistic IoT configuration, so the prediction can be completed inside the coherence time it is meant to anticipate. The controller itself is a table lookup over |K|×Q=72 entries and takes 71 μsin its unoptimized Python implementation.

### 7.13. Practical Implications and Deployment Considerations

The study isolates a single link so that the effect of CSI age can be measured cleanly. The following paragraphs discuss how the framework carries over to the conditions of real IoT deployments.

When many nodes share a bandwidth budget, the rate decisions interact. Because the belief-averaged distortion ${\bar{D}}_{u}\left(k\right)=E_{{\hat{F}}_{u,n}}\left[D\left(k,\gamma \right)\right]$ of each user *u* is computed separately, the per-user rule (10) extends naturally to a shared budget $\sum_{u}k_{u}\leq K_{\mathrm{tot}}$ through Lagrangian relaxation: each user selects $\arg\min_{k}\left\{{\bar{D}}_{u}\left(k\right)+\lambda k\right\}$, and a scalar price λ is adjusted until the budget is met. The complexity stays linear in the number of users, and the curvature argument of Proposition 1 applies user by user. Two further effects arise when the link is part of a shared network. Scheduling and interference make the SNR law of one user depend on the decisions made for others, so the predictor would need interference measurements as additional inputs, and a shared budget couples the errors of different users, so the gain of belief averaging for the network as a whole can be smaller or larger than for an isolated link. Multi-user semantic transmission has been studied with contemporaneous channel knowledge [31]; combining such designs with outdated CSI is a natural next step. The multi-user extension outlined here has not yet been validated experimentally; all experiments in this paper concern a single point-to-point link.

With receive diversity or precoding, the quantity that drives the codec is the post-processing SNR. The belief can be defined on that scalar, and the controller is unchanged. Two consequences follow. Diversity reduces the variance of the effective SNR, which, by Proposition 1, reduces the plug-in penalty in quasi-static conditions. Precoding, on the other hand, is far more sensitive to CSI age than a scalar SNR, because the phase of the channel vector also ages [15]; the predictor would then have to forecast the channel vector, and its input and output dimensions grow with the number of antennas. Deep JSCC schemes that adapt to MIMO channel matrices [32] could take such a predictive law as their conditioning input. As with the multi-user case, this multi-antenna extension has not yet been validated experimentally in this study.

On a frequency-selective channel observed through many subcarriers, a single effective SNR summarizes the link for the codec, and the belief and the controller are defined on that scalar without change. Frequency diversity makes this quantity less variable than the SNR of a flat channel, which leaves less room for any adaptive scheme, but it does not remove the effect of aged reports: at high mobility, adaptation on outdated reports again performs worse than the fixed-rate link, and prediction brings it back (Section 7.9). Codecs that allocate symbols to individual subcarriers would instead need a forecast of the per-subcarrier gains, which the same quantile construction could provide with a vector output.

For dynamically changing environments, several results of this study are directly relevant. A single predictor trained over the whole mobility range retains most of the benefit of regime-specific training, and a predictor trained for one regime recovers nearly all of it after a few seconds of fine-tuning on a few thousand reports from the new regime (Section 7.7). When the mobility drifts within a record or the estimation quality changes over time, the learned predictor keeps its advantage over outdated CSI without retraining (Section 7.9), and on measured logs a predictor trained on earlier recordings generalizes to new drives and static sessions (Section 7.8). When conditions drift beyond the training range, the coverage of the belief can be monitored from recent feedback at negligible cost, and the offsets of Section 4.2 can be re-estimated from that same feedback. The measured logs show that such offsets must come from the deployment itself: offsets taken from other recordings increased the coverage error.

If the codec or the source changes, only the distortion table needs attention: it belongs to a specific codec and source. If either changes, the table must be measured again; for the codec used here this takes a few minutes on a CPU and does not involve the predictor. The controller and the predictor remain unchanged.

In terms of signaling overhead, the scheme uses only the CSI that an adaptive link already feeds back; the window of *W* past values is buffered at the transmitter, and the inference cost reported in Table 10, a few milliseconds per block for encoder, predictor and controller together on a single CPU thread, fits inside block intervals of that order or longer.

## 8. Conclusions

This study shows that delayed CSI is not a minor implementation detail in rate-adaptive semantic communication. As channel aging increases, a controller that reacts to stale measurements can lose the benefit of adaptation and, in the tested high-mobility regimes, become less reliable than a bandwidth-matched fixed-rate link. The same trend appears in SNR logs measured in a commercial 5G/LTE network, where the advantage of outdated-CSI adaptation over a fixed-rate link fades as the feedback ages, so the effect is not an artifact of a fading model. The proposed remedy combines horizon-matched channel prediction with a distribution-aware rate decision. It can be added to an existing adaptive codec because it operates on the measured distortion surface rather than changing the encoder or decoder architecture.

The results also clarify where the improvement comes from. Prediction recovers part of the information lost during feedback delay: close to half of the outdated-CSI penalty at high mobility in simulation, and a smaller but consistent part on measured logs, where much of the second-scale SNR variation cannot be anticipated from its past. A single predictor trained over the whole mobility range retains most of this benefit without retraining, a predictor trained for one regime can be fine-tuned to another within seconds, and the benefit over outdated CSI persists under drifting mobility, varying estimation quality and frequency-selective multipath. Belief averaging accounts for the asymmetric cost created by a convex distortion response, whose curvature assumptions were verified on the trained codec. At the boundaries between discrete rates, the same analysis predicts how often uncertainty changes the chosen rate, and the measured switching frequency agrees with that prediction. When all schemes are held to the same average bandwidth, belief and point decisions from the same predictor differ only slightly on average; the belief matters most when decisions fall on the steep part of the surface and when the forecast uncertainty differs from block to block. The number and placement of the quantile levels have little influence, and the pinball-trained quantiles need no further recalibration. At the task level, the clearest effect is the loss that outdated CSI causes in a level-crossing alarm and its removal by prediction, while the differences among the prediction-based schemes remain small. Under matched Clarke statistics, the genie-aided Wiener predictor remains the strongest baseline. Under Doppler mismatch, however, the learned predictor is more robust because its uncertainty is estimated from the deployment distribution. Taken together, these findings do not support a general claim that learning outperforms model-based estimation; they show instead that a learned predictor is the safer choice when the channel statistics are uncertain.

Several directions follow naturally. The first is an experimental validation of the multi-user and multi-antenna extensions discussed in Section 7.13. Shared-resource scheduling can build on the per-user belief-averaged distortion through a price on bandwidth; multi-antenna links require predictors of the effective post-processing SNR or of the channel vector itself; subcarrier-level resource allocation would call for vector-valued forecasts; stream-specific distortion tables would let the controller exploit the large differences between sensor types; and budget-constrained controllers that minimize distortion for a given average bandwidth would extend the framework to low-fidelity operating points. The same curvature-aware principle may also be useful for power, scheduling or beam decisions made from uncertain future CSI.

## Figures and Tables

**Figure 1:**
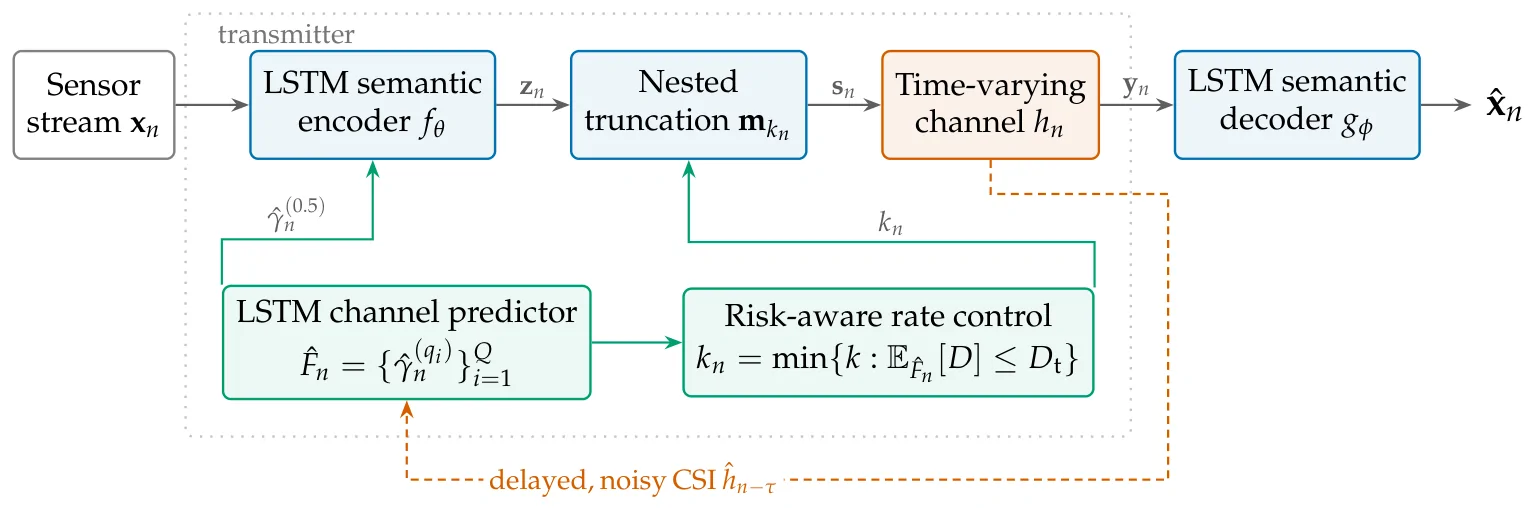
Prediction-aided adaptive semantic communication. The transmitter observes the delayed, noisy CSI ${\hat{h}}_{n-\tau }$, from which an LSTM predictor produces a predictive law ${\hat{F}}_{n}$ for the receive SNR $\gamma_{n}$, represented by *Q* quantiles. The rate controller averages the measured distortion surface over ${\hat{F}}_{n}$ to choose the rate $k_{n}$, and the predictive median ${\hat{\gamma }}_{n}^{\left(0.5\right)}$ conditions the semantic encoder.

**Figure 2:**
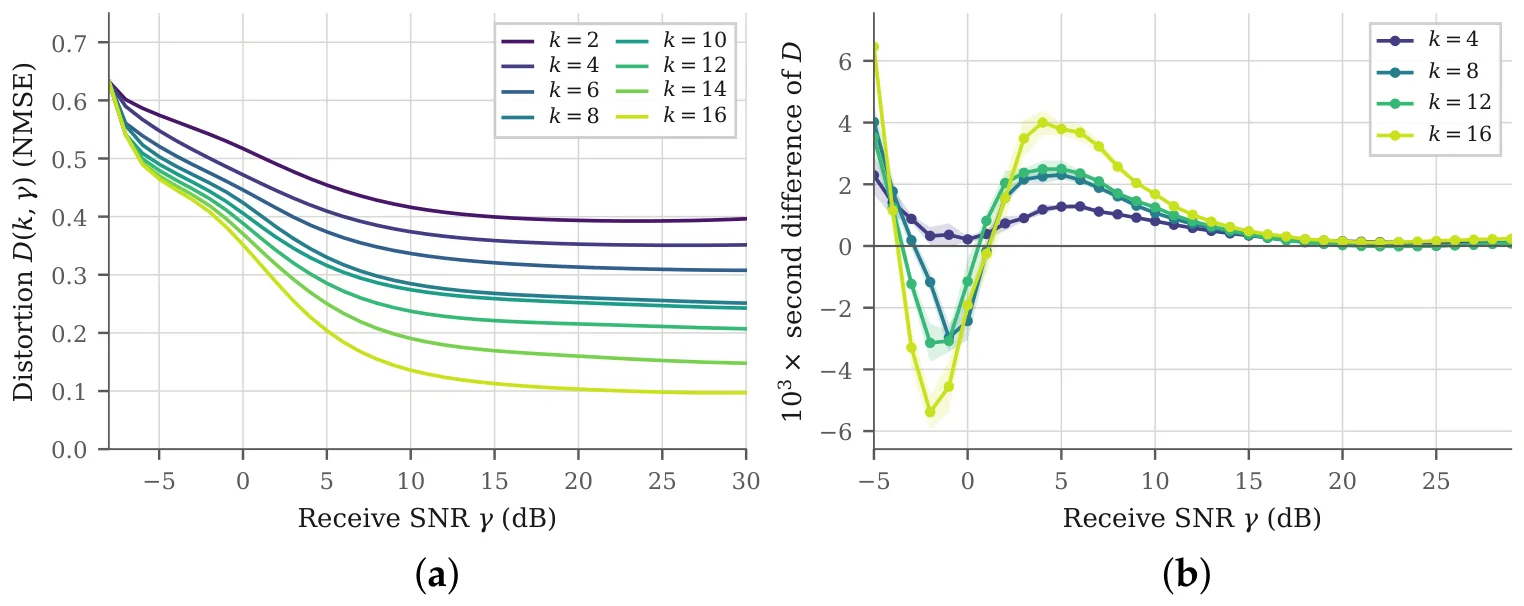
(**a**) Measured distortion surface D(k,γ) of the trained semantic codec. (**b**) Second difference of D(k,γ) on the 1 dB grid for k∈{4,8,12,16}, with bands of ±2 standard errors.

**Figure 3:**
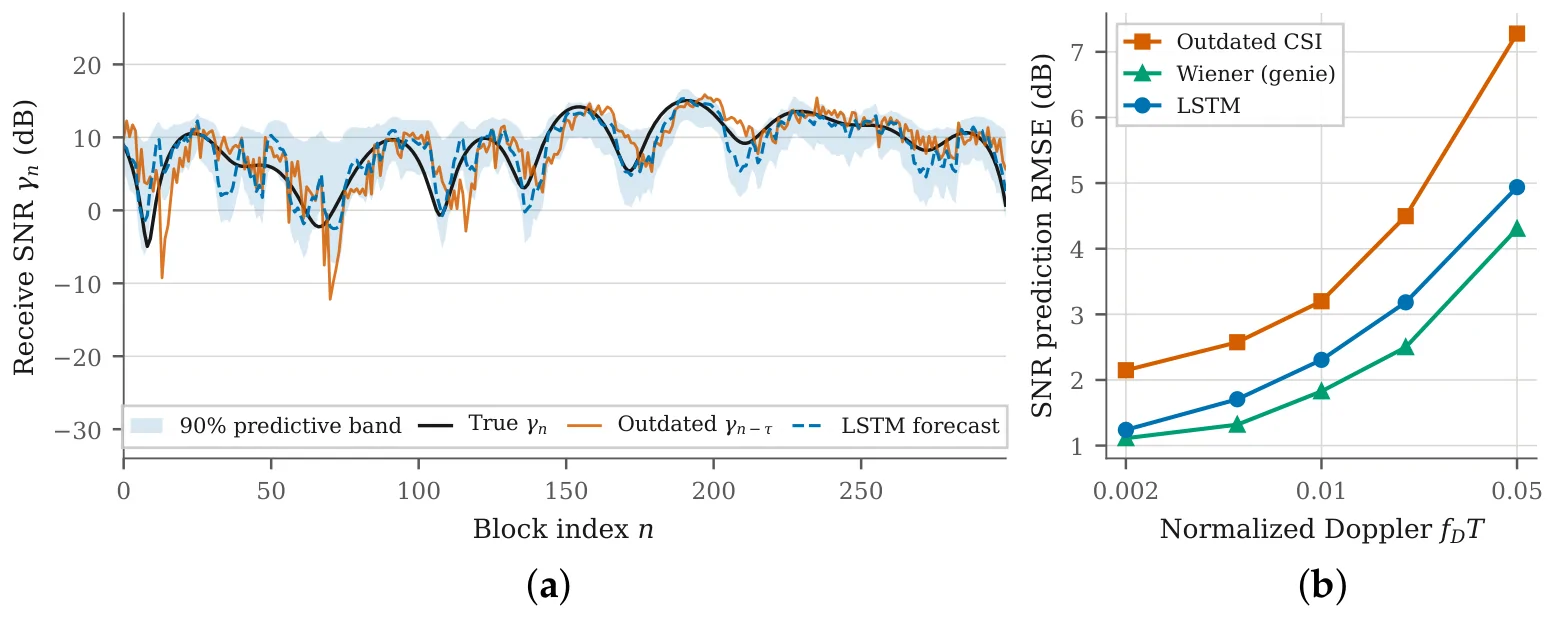
(**a**) Receive SNR: true process, outdated surrogate, LSTM point forecast and 90% predictive band. (**b**) SNR prediction RMSE versus normalized Doppler on the test realizations.

**Figure 4:**
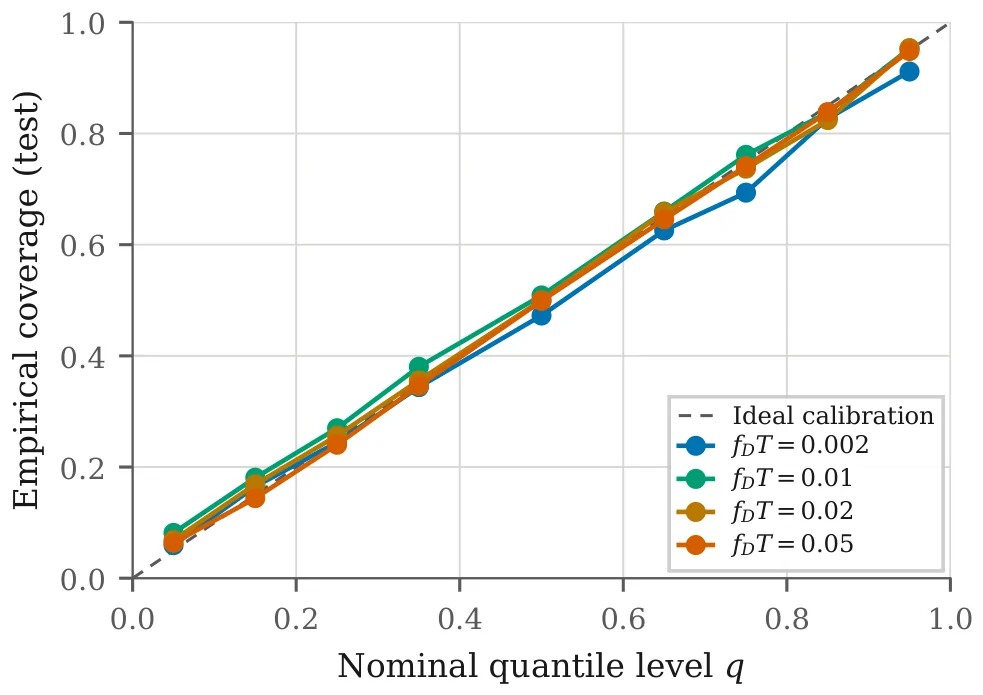
Reliability diagram of the predictive quantiles on the test realizations.

**Figure 5:**
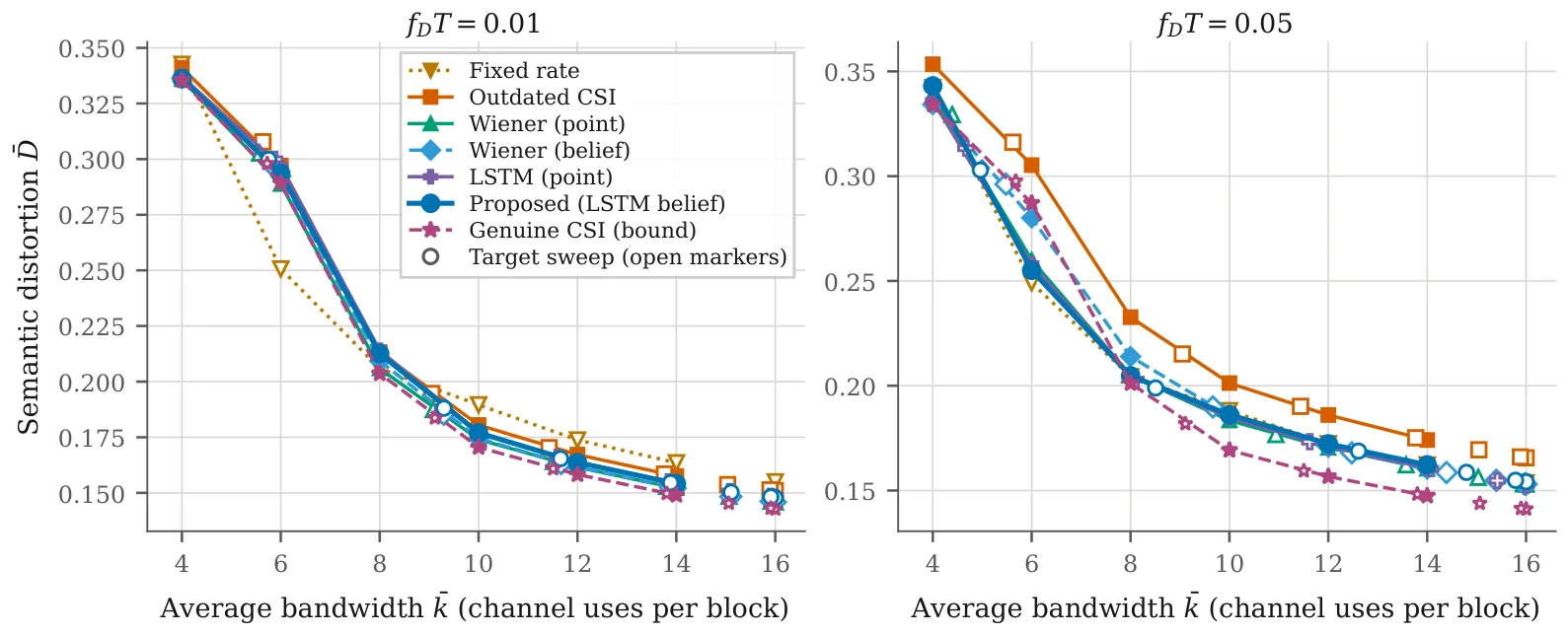
Averagebandwidth versus semantic distortion at $f_{D}T=0.01$ (**left**) and $f_{D}T=0.05$ (**right**), τ=5; lines: all schemes matched to each average bandwidth; open markers: operating points obtained directly for each fidelity target.

**Figure 6:**
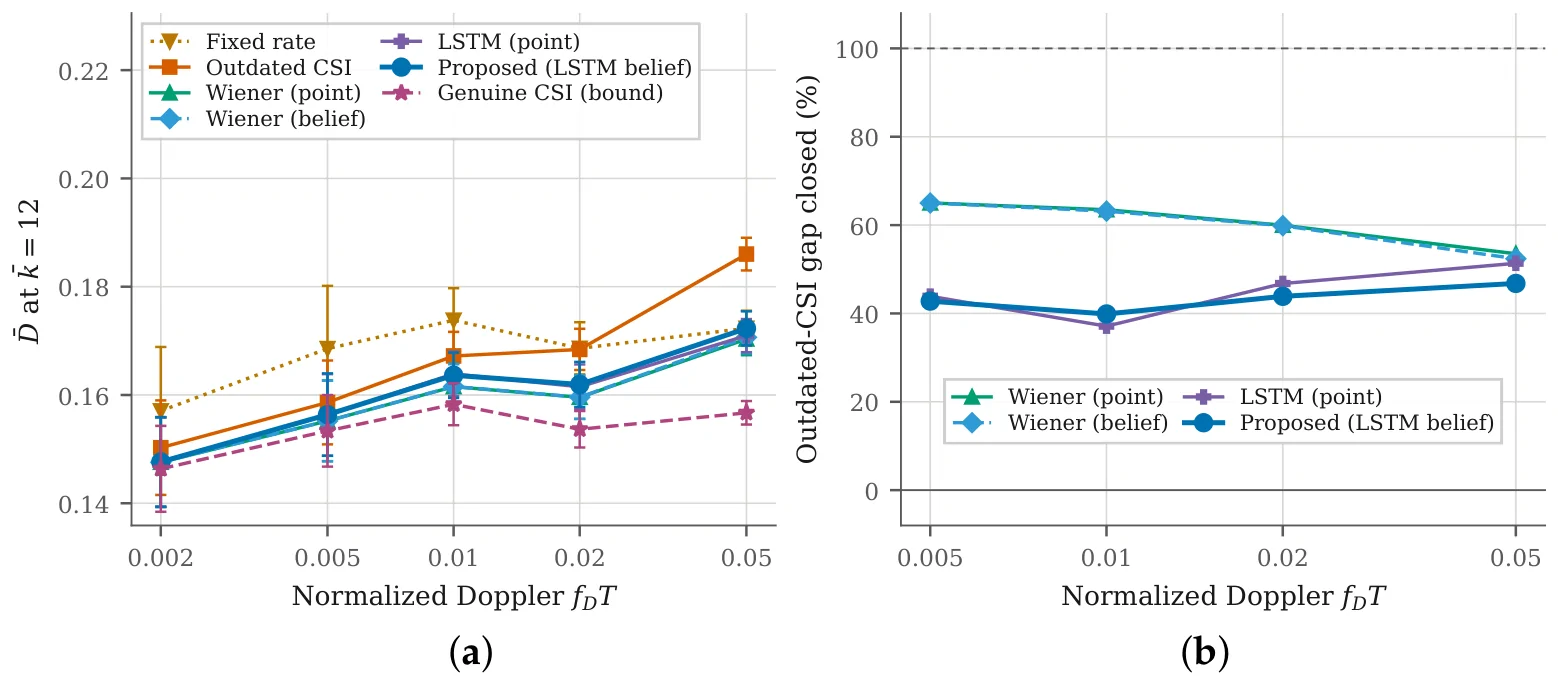
(**a**) Semantic distortion at a matched average bandwidth of $\bar{k}=12$ versus normalized Doppler. (**b**) Fraction of the outdated-CSI penalty recovered by each predictor; 100% corresponds to the genuine-CSI bound.

**Figure 7:**
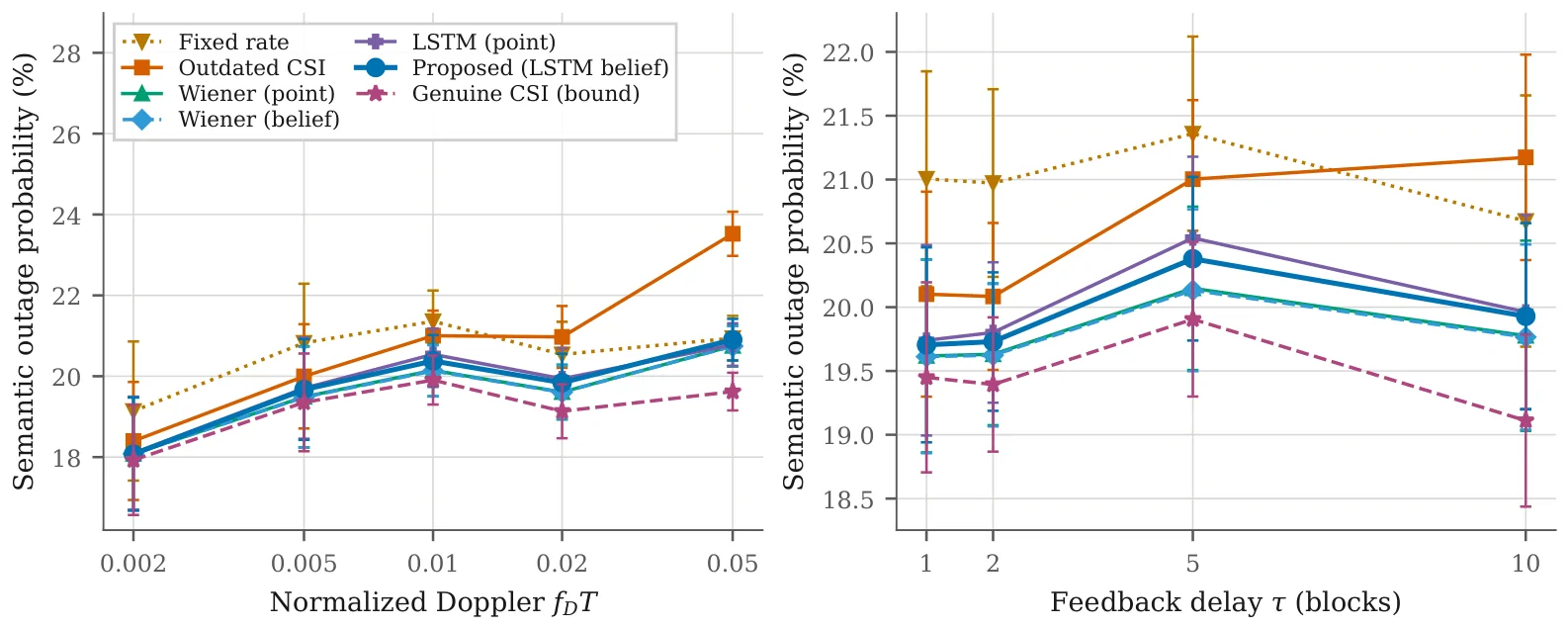
Semantic outage probability at $\bar{k}=12$ ($D_{\mathrm{req}}=0.25$, 95% confidence intervals) versus normalized Doppler (**left**, τ=5) and versus feedback delay (**right**, $f_{D}T=0.01$).

**Figure 8:**
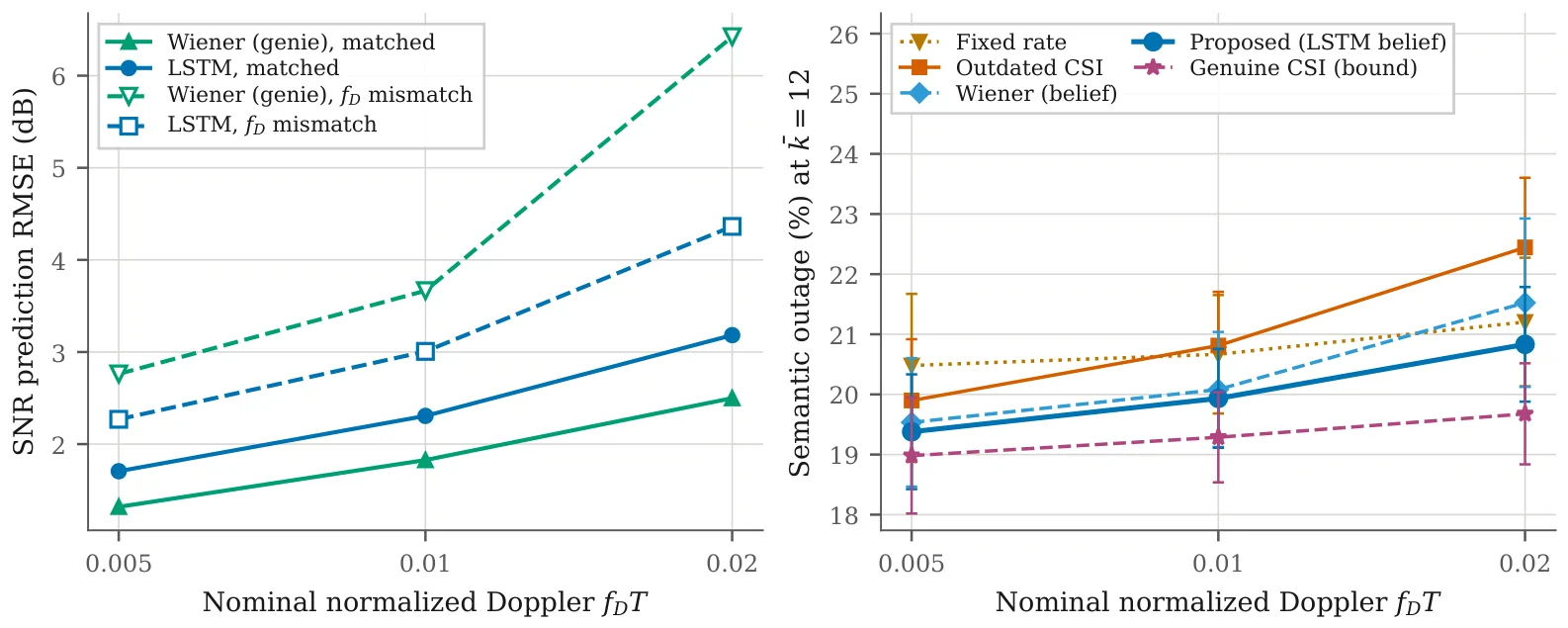
Robustness to model mismatch. (**Left**): SNR prediction RMSE under the matched Clarke process (solid) and under the Doppler mismatch, where the maximum Doppler is unknown to within a factor of four (dashed, open markers). (**Right**): Semantic outage at a matched average bandwidth of $\bar{k}=12$ under the Doppler mismatch, with 95% confidence intervals.

**Figure 9:**
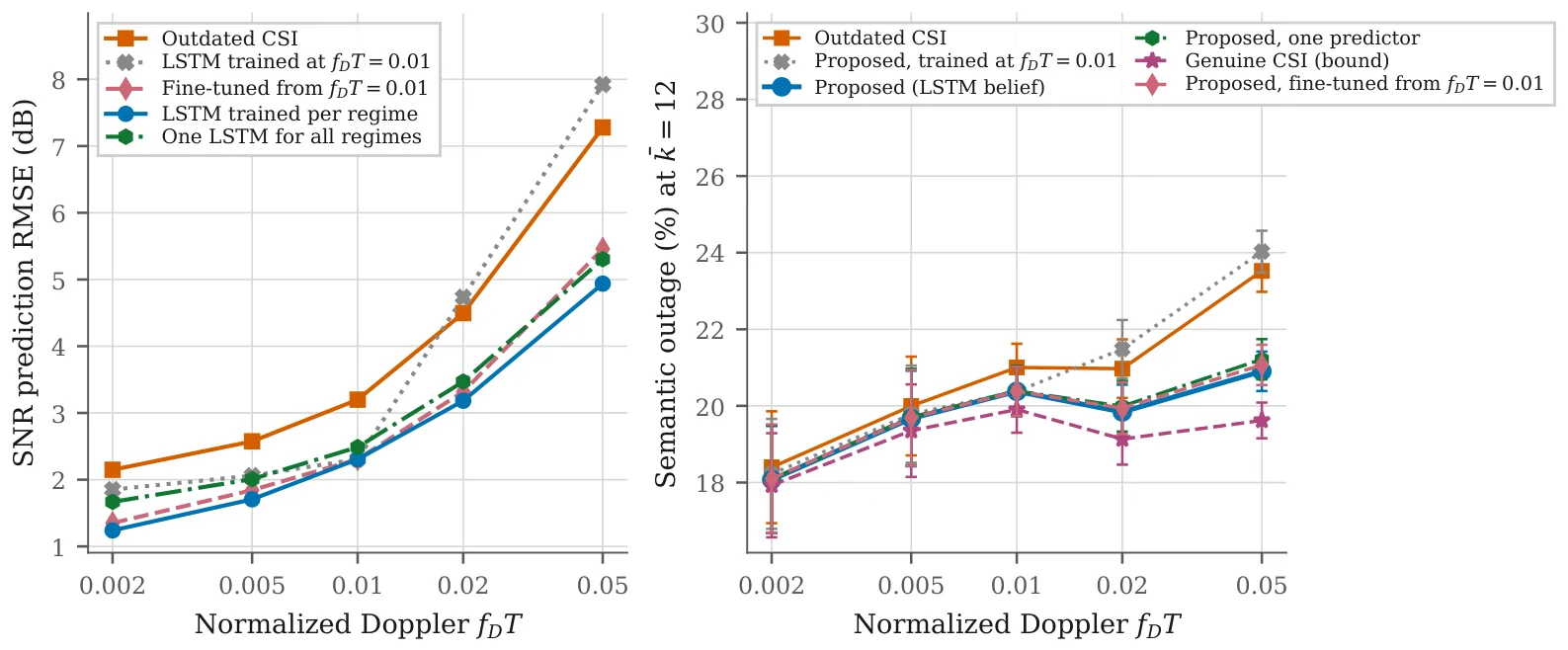
Generalization and adaptation across mobility regimes. (**Left**): SNR prediction RMSE versus normalized Doppler for the outdated CSI, a predictor trained only at $f_{D}T=0.01$, the same predictor fine-tuned on a small adaptation set from each regime, regime-specific predictors and one mobility-agnostic predictor. (**Right**): Semantic outage at $\bar{k}=12$, τ=5, with 95% confidence intervals.

**Figure 10:**
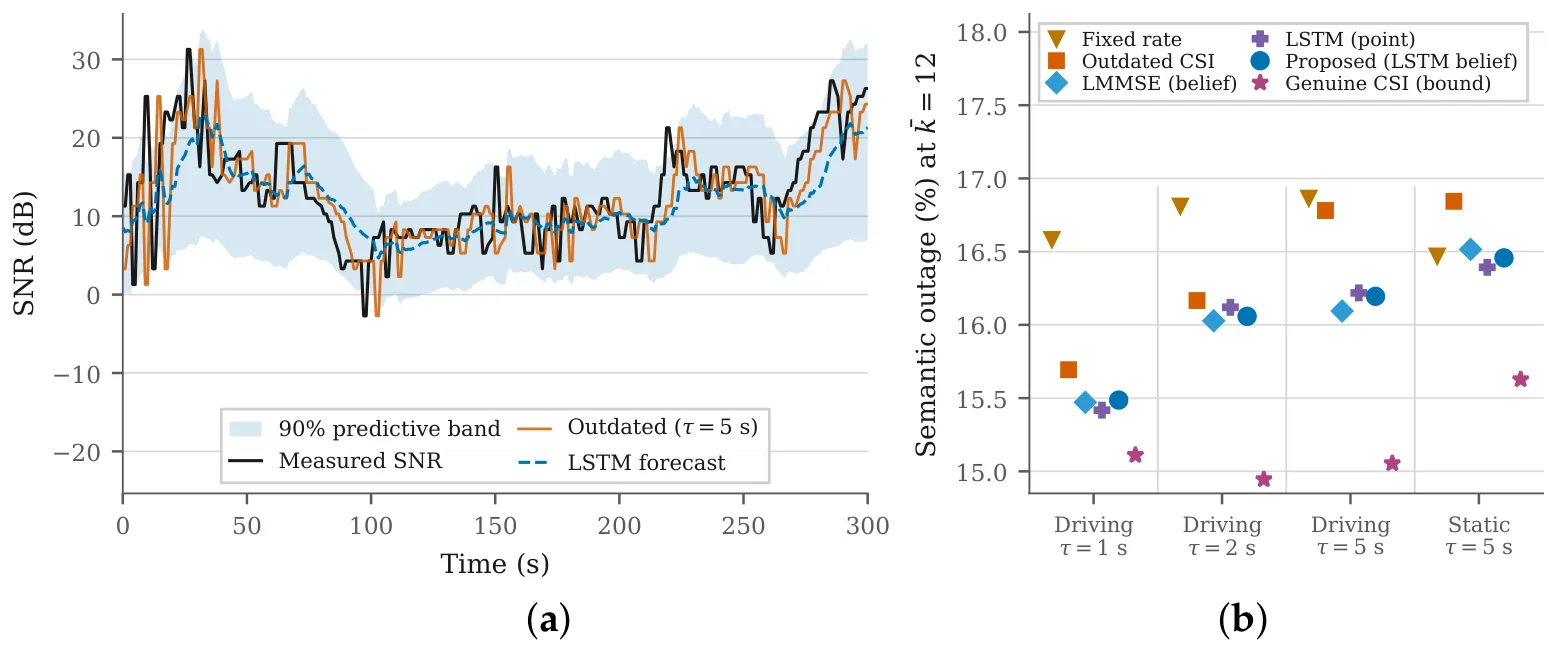
Measured 5G/LTE channel logs. (**a**) Excerpt of a driving test log: Measured SNR, outdated value, LSTM forecast and 90% predictive band. (**b**) Semantic outage at $\bar{k}=12$ for driving logs with τ∈{1,2,5} s and static logs with τ=5 s (block-weighted means over the test logs).

**Figure 11:**
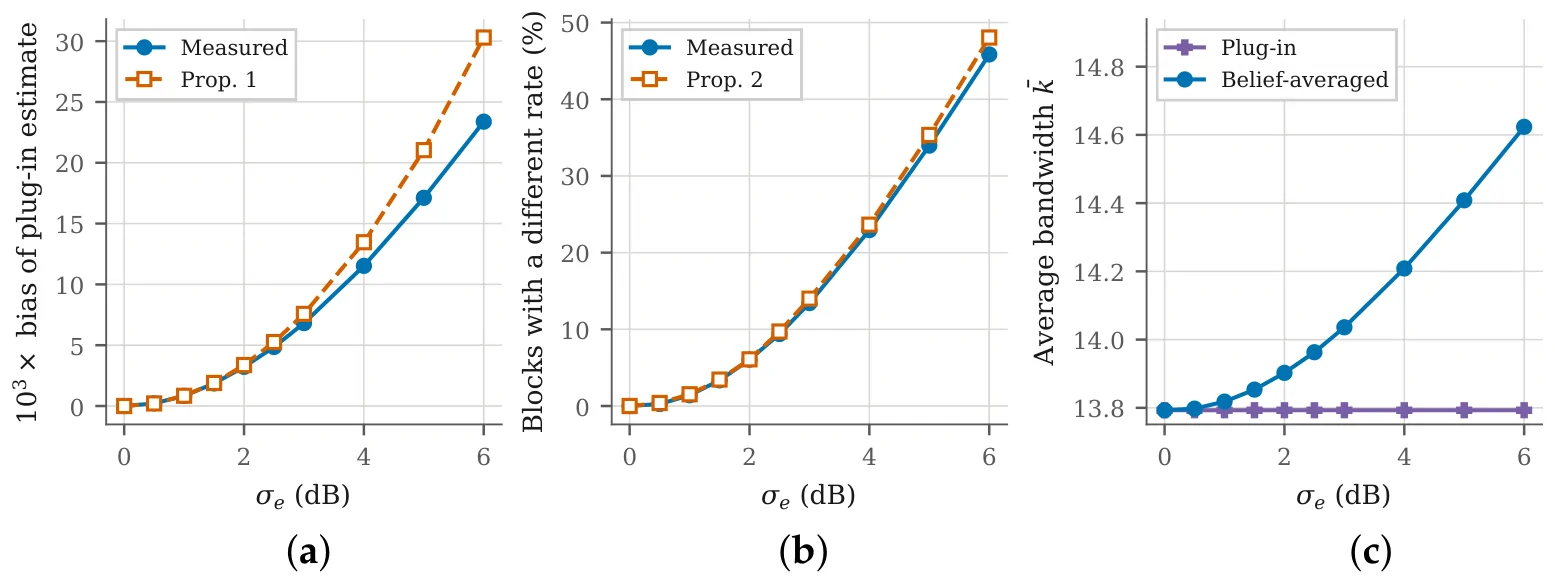
Numerical illustration of Propositions 1 and 2, averaged over operating points with $\hat{\gamma }\geq 0$ dB. (**a**) Bias of the plug-in distortion estimate and its second-order prediction. (**b**) Fraction of operating points at which the belief-averaged and plug-in rules select different rates, measured and predicted by (17). (**c**) Average bandwidth spent by the two controllers at a fidelity target of 0.24.

**Table 1:** Comparison with related adaptive semantic communication and channel prediction studies.

References	Rate Adaptive	Delayed CSI	Channel Forecast	Predictive Law Used	Error-to- Distortion Analysis	Real Source Data	Measured Channel Traces
ADJSCC [12]	SNR-cond.	–	–	–	–	✓	–
DeepJSCC-f [14]	feedback	–	–	–	–	✓	–
Bandwidth-agile [26]	✓	–	–	–	–	✓	–
Rate-adaptive [13]	✓	–	–	–	–	✓	–
PADC [18]	✓	–	–	–	–	✓	–
Fading prediction [27]	–	✓	✓	–	–	–	–
**This work**	✓	✓	✓	✓	✓	✓	✓

**Table 2:** Datasets and their roles.

Data	Role	Size	Used for
NAB sensor blocks	training	9545 blocks	codec weights
	validation	2317 blocks	distortion table, detector
	test	1964 blocks	end-to-end evaluation only
Simulated channel (per $f_{D}T$, τ, regime)	training	24 seq. × 900 blocks ^†^	predictor weights
	validation	6 seq. × 900 blocks	epoch selection
	calibration	6 seq. × 900 blocks	coverage check, optional offsets
	test	20 seq. × 1964 blocks	end-to-end evaluation only
	adaptation	4 seq. × 900 blocks	fine-tuning (Section 7.7)
Measured 5G/LTE logs (driving/static)	training	36/15 logs	predictor and LMMSE weights
	validation	6/2 logs	epoch selection
	calibration	6/2 logs	coverage check, optional offsets
	test	11/4 logs	end-to-end evaluation only

^†^ 40 sequences for the Doppler-mismatch mixture and 60 for the mobility-agnostic predictors. The adaptation sequences come from a separate random stream and never overlap with the test realizations.

**Table 3:** Accuracy and calibration of the predictive laws on the test data (ACE: average coverage error; PICP_90_ and Width_90_: coverage and mean width of the central 90% interval). The model-based belief is the exact law of the genie-aided Wiener filter for simulated channels and the Gaussian LMMSE law for measured logs.

		LSTM Quantile Head					Model-Based Belief	
Setting	RMSE (dB)	ACE	PICP_90_	Width_90_ (dB)	Pinball (dB)	ACE, Recal.	ACE	PICP_90_
$f_{D}T=0.002$	1.24	0.023	0.852	2.4	0.24	0.024	0.003	0.903
$f_{D}T=0.005$	1.71	0.012	0.866	3.6	0.34	0.026	0.023	0.908
$f_{D}T=0.01$	2.31	0.018	0.871	5.3	0.50	0.009	0.027	0.906
$f_{D}T=0.02$	3.18	0.011	0.885	8.5	0.75	0.017	0.024	0.902
$f_{D}T=0.05$	4.94	0.007	0.885	15.1	1.29	0.004	0.011	0.895
Shape mismatch, 0.02	2.06	0.012	0.892	5.7	0.48	0.006	0.124	0.902
Doppler mismatch, 0.02	4.36	0.011	0.861	11.5	1.06	0.010	0.131	0.523
Measured, driving, τ=5 s	6.34	0.036	0.836	17.7	1.71	0.078	0.025	0.818
Measured, static, τ=5 s	3.72	0.018	0.940	14.6	1.01	0.066	0.056	0.966

**Table 4:** Semantic distortion and outage at $\bar{k}=12$ (mean ± 95% confidence interval over 20 test realizations). ^∘^: Difference from the proposed scheme not significant (paired Wilcoxon test, p≥0.05); all other differences are significant.

	$f_{D}T=0.02$		$f_{D}T=0.05$		Doppler Mismatch, $f_{D}T=0.02$	
Scheme	$\bar{D}$	$P_{\mathrm{out}}$ (%)	$\bar{D}$	$P_{\mathrm{out}}$ (%)	$\bar{D}$	$P_{\mathrm{out}}$ (%)
Fixed rate	0.1686 ± 0.0048	20.54 ± 0.81	0.1723 ± 0.0032 ^∘^	20.94 ± 0.55 ^∘^	0.1725 ± 0.0081 ^∘^	21.20 ± 1.07 ^∘^
Outdated CSI	0.1684 ± 0.0038	20.97 ± 0.76	0.1860 ± 0.0030	23.52 ± 0.55	0.1772 ± 0.0080	22.44 ± 1.16
Wiener (point)	0.1596 ± 0.0040	19.61 ± 0.68	0.1703 ± 0.0030	20.75 ± 0.51 ^∘^	0.1740 ± 0.0100 ^∘^	21.49 ± 1.34 ^∘^
Wiener (belief)	0.1596 ± 0.0040	19.60 ± 0.66	0.1707 ± 0.0030	20.75 ± 0.49 ^∘^	0.1743 ± 0.0105 ^∘^	21.52 ± 1.40 ^∘^
LSTM (point)	0.1615 ± 0.0041	19.94 ± 0.75 ^∘^	0.1710 ± 0.0031	20.78 ± 0.53	0.1682 ± 0.0071	20.85 ± 0.99 ^∘^
Proposed	0.1620 ± 0.0042	19.84 ± 0.71	0.1723 ± 0.0031	20.90 ± 0.52	0.1690 ± 0.0071	20.83 ± 0.95
Genuine CSI (bound)	0.1537 ± 0.0034	19.13 ± 0.67	0.1567 ± 0.0022	19.62 ± 0.47	0.1562 ± 0.0055	19.68 ± 0.84

**Table 5:** Per-stream semantic distortion and outage at $f_{D}T=0.05$, τ=5, $\bar{k}=12$ (mean ± 95% confidence interval over 20 test realizations; Blocks is the number of test blocks per realization).

Stream	Blocks	Fixed	Outdated	Wiener (b.)	LSTM (p.)	Proposed	Genuine
Semantic distortion $\bar{D}$							
Machine temperature	702	0.067 ± 0.004	0.073 ± 0.004	0.064 ± 0.004	0.066 ± 0.004	0.066 ± 0.004	0.059 ± 0.003
Ambient temperature	220	0.066 ± 0.004	0.071 ± 0.003	0.065 ± 0.003	0.065 ± 0.003	0.065 ± 0.004	0.060 ± 0.003
CPU utilization	557	0.259 ± 0.013	0.284 ± 0.012	0.258 ± 0.011	0.257 ± 0.012	0.260 ± 0.012	0.227 ± 0.008
Request latency	119	0.640 ± 0.011	0.665 ± 0.008	0.653 ± 0.012	0.643 ± 0.010	0.645 ± 0.010	0.645 ± 0.007
NYC taxi demand	315	0.111 ± 0.008	0.122 ± 0.007	0.103 ± 0.007	0.107 ± 0.007	0.109 ± 0.008	0.089 ± 0.005
Keystroke timing	51	0.426 ± 0.016	0.452 ± 0.014	0.432 ± 0.013	0.431 ± 0.013	0.430 ± 0.014	0.431 ± 0.010
Semantic outage $P_{\mathrm{out}}$ (%)							
Machine temperature	702	4.4 ± 0.6	5.1 ± 0.6	3.9 ± 0.5	4.1 ± 0.6	4.2 ± 0.6	3.1 ± 0.5
Ambient temperature	220	2.5 ± 0.6	3.1 ± 0.7	2.1 ± 0.6	2.3 ± 0.7	2.4 ± 0.7	1.4 ± 0.5
CPU utilization	557	33.2 ± 2.0	40.1 ± 2.0	34.2 ± 1.8	33.3 ± 1.9	33.5 ± 1.9	32.4 ± 1.7
Request latency	119	100.0 ± 0.0	100.0 ± 0.0	100.0 ± 0.0	100.0 ± 0.0	100.0 ± 0.0	100.0 ± 0.0
NYC taxi demand	315	8.0 ± 1.3	9.7 ± 1.1	6.5 ± 1.0	7.5 ± 1.1	7.8 ± 1.2	4.6 ± 0.8
Keystroke timing	51	89.6 ± 1.2	90.5 ± 1.0	88.8 ± 1.3	90.1 ± 1.2	89.4 ± 1.0	90.4 ± 1.2

**Table 6:** Resultson the measured 5G/LTE test logs at $\bar{k}=12$ (block-weighted mean ± half-width of the 95% log-level bootstrap interval). ^∘^: Not significantly different from the proposed scheme (paired Wilcoxon test over logs, p≥0.05).

	Driving, τ=1 s		Driving, τ=2 s		Driving, τ=5 s		Static, τ=5 s	
Scheme	$\bar{D}$	$P_{\mathrm{out}}$ (%)	$\bar{D}$	$P_{\mathrm{out}}$ (%)	$\bar{D}$	$P_{\mathrm{out}}$ (%)	$\bar{D}$	$P_{\mathrm{out}}$ (%)
Fixed rate	0.1414 ± 0.0231	16.6 ± 4.2	0.1413 ± 0.0242	16.8 ± 4.4	0.1423 ± 0.0250	16.9 ± 4.6	0.1365 ± 0.0213 ^∘^	16.5 ± 3.2 ^∘^
Outdated CSI	0.1300 ± 0.0198	15.7 ± 4.0	0.1347 ± 0.0213	16.2 ± 4.1 ^∘^	0.1386 ± 0.0234	16.8 ± 4.4	0.1380 ± 0.0170 ^∘^	16.8 ± 2.9 ^∘^
LMMSE (point)	0.1301 ± 0.0198 ^∘^	15.5 ± 3.9 ^∘^	0.1338 ± 0.0210 ^∘^	16.1 ± 4.1 ^∘^	0.1357 ± 0.0227 ^∘^	16.1 ± 4.4 ^∘^	0.1355 ± 0.0164 ^∘^	16.5 ± 2.7 ^∘^
LMMSE (belief)	0.1301 ± 0.0199 ^∘^	15.5 ± 3.9 ^∘^	0.1340 ± 0.0212 ^∘^	16.0 ± 4.1 ^∘^	0.1359 ± 0.0228 ^∘^	16.1 ± 4.4 ^∘^	0.1356 ± 0.0152 ^∘^	16.5 ± 2.6 ^∘^
LSTM (point)	0.1299 ± 0.0197 ^∘^	15.4 ± 3.9 ^∘^	0.1339 ± 0.0212 ^∘^	16.1 ± 4.1 ^∘^	0.1359 ± 0.0229 ^∘^	16.2 ± 4.4 ^∘^	0.1354 ± 0.0170 ^∘^	16.4 ± 2.8 ^∘^
Proposed	0.1297 ± 0.0198	15.5 ± 4.0	0.1335 ± 0.0209	16.1 ± 4.1	0.1359 ± 0.0229	16.2 ± 4.4	0.1356 ± 0.0163	16.5 ± 2.7
Genuine CSI (bound)	0.1250 ± 0.0193	15.1 ± 3.9	0.1248 ± 0.0201	14.9 ± 4.1	0.1256 ± 0.0213	15.1 ± 4.3	0.1298 ± 0.0115 ^∘^	15.6 ± 2.3 ^∘^
SNR RMSE (dB): outdated/LMMSE/LSTM	4.39/4.04/3.97		6.28/5.56/5.54		7.40/6.38/6.34		4.83/3.78/3.72	

**Table 7:** Resultsunder non-stationary Doppler, time-varying CSI quality and frequency-selective multipath at $\bar{k}=12$, τ=5 (mean ± 95% confidence interval over 20 test realizations). ^†^ Wiener filter designed for $f_{D}T=0.01$ (non-stationary Doppler) or $f_{D}T=0.02$ (time-varying CSI quality); LMMSE predictor on the effective-SNR reports (frequency-selective). Proposed: Mobility-agnostic predictor (non-stationary Doppler), predictor trained at $f_{D}T=0.02$ (time-varying CSI quality) and scalar-input predictor (frequency-selective). ^∘^: Not significantly different from the proposed scheme (paired Wilcoxon test, p≥0.05).

	Non-Stationary Doppler		Time-Varying CSI Quality		Freq.-Selective $f_{D}T=0.02$		Freq.-Selective $f_{D}T=0.05$	
Scheme	$\bar{D}$	$P_{\mathrm{out}}$ (%)	$\bar{D}$	$P_{\mathrm{out}}$ (%)	$\bar{D}$	$P_{\mathrm{out}}$ (%)	$\bar{D}$	$P_{\mathrm{out}}$ (%)
Fixed rate	0.1684 ± 0.0077	20.7 ± 1.2	0.1676 ± 0.0048	20.6 ± 0.6	0.1341 ± 0.0017	16.4 ± 0.5	0.1328 ± 0.0011	16.3 ± 0.2 ^∘^
Outdated CSI	0.1676 ± 0.0063	21.0 ± 1.2	0.1700 ± 0.0039	21.3 ± 0.6	0.1325 ± 0.0012	16.0 ± 0.3	0.1355 ± 0.0011	16.6 ± 0.2
Model-based (point) ^†^	0.1676 ± 0.0074	20.8 ± 1.2	0.1612 ± 0.0038	19.9 ± 0.6 ^∘^	0.1320 ± 0.0012	16.0 ± 0.4	0.1342 ± 0.0010	16.4 ± 0.2 ^∘^
Model-based (belief) ^†^	0.1677 ± 0.0074	20.8 ± 1.2	0.1613 ± 0.0037	19.9 ± 0.6	0.1320 ± 0.0012	15.9 ± 0.4	0.1342 ± 0.0010	16.4 ± 0.2 ^∘^
LSTM (point)	0.1622 ± 0.0063 ^∘^	20.1 ± 1.1	0.1632 ± 0.0039 ^∘^	20.3 ± 0.6	0.1317 ± 0.0013 ^∘^	15.8 ± 0.4 ^∘^	0.1333 ± 0.0011 ^∘^	16.3 ± 0.2
Proposed	0.1624 ± 0.0063	20.0 ± 1.1	0.1632 ± 0.0041	20.0 ± 0.6	0.1317 ± 0.0012	15.8 ± 0.4	0.1333 ± 0.0011	16.3 ± 0.2
Genuine CSI (bound)	0.1539 ± 0.0049	19.2 ± 0.9	0.1542 ± 0.0029	19.3 ± 0.6	0.1308 ± 0.0009	15.7 ± 0.3	0.1302 ± 0.0008	15.7 ± 0.2
SNR RMSE (dB): outdated/ model-based/LSTM	4.50/5.03/3.40		4.79/2.83/3.51		1.86/1.59/1.29		3.67/3.08/2.74	

**Table 8:** Sensitivity to the quantile representation and to the decision criterion (τ=5). ^∘^: Not significantly different from the default proposed scheme (Q=9, default levels), paired Wilcoxon test, p≥0.05.

	$f_{D}T=0.02$			$f_{D}T=0.05$		
Variant	$\bar{D}$, $\bar{k}=12$	$P_{\mathrm{out}}$, $\bar{k}=12$	$\bar{D}$, $\bar{k}=8$	$\bar{D}$, $\bar{k}=12$	$P_{\mathrm{out}}$, $\bar{k}=12$	$\bar{D}$, $\bar{k}=8$
*Number and placement of quantile levels*						
Q=1 (median only)	0.1617	19.87 ^∘^	0.2054 ^∘^	0.1718	20.81	0.2046 ^∘^
Q=3, equal mass	0.1618 ^∘^	19.85 ^∘^	0.2048 ^∘^	0.1723 ^∘^	20.89 ^∘^	0.2051
Q=5, equal mass	0.1619 ^∘^	19.95	0.2062	0.1721	20.87 ^∘^	0.2055
Q=9, default levels	0.1620	19.84	0.2053	0.1723	20.90	0.2047
Q=9, equal mass	0.1620 ^∘^	19.85 ^∘^	0.2048	0.1719	20.85 ^∘^	0.2057
Q=19, equal mass	0.1618	19.87 ^∘^	0.2066	0.1721	20.85 ^∘^	0.2058
Q=9, lower-tail dense	0.1612	19.90 ^∘^	0.2050 ^∘^	0.1713	20.80 ^∘^	0.2031
*Decision criterion*						
Point head (plug-in)	0.1615	19.94 ^∘^	0.2079	0.1710	20.78	0.2048 ^∘^
With recalibration	0.1618	19.87 ^∘^	0.2055	0.1724	20.90 ^∘^	0.2048 ^∘^
Quantile rule (11), ε=0.15	0.1621 ^∘^	19.88 ^∘^	0.2484	0.1736	21.13	0.2164
Quantile rule (11), ε=0.05	0.1622 ^∘^	19.89 ^∘^	0.2455	0.1814	22.85	0.2375

**Table 9:** Task-level results at $f_{D}T=0.05$, τ=5 (mean ± 95% confidence interval over 20 test realizations). Anomaly detection is reported at $\bar{k}=12$; its clean-source $F_{1}$ is 0.489. ^∘^: Not significantly different from the proposed scheme.

	Anomaly Detection		Level-Crossing Alarm ($F_{1}$)	
Scheme	$F_{1}$	Decision Agreement	$\bar{k}=12$	$\bar{k}=8$
Fixed rate	0.473 ± 0.003	0.907 ± 0.003 ^∘^	0.487 ± 0.007	0.432 ± 0.007
Outdated CSI	0.473 ± 0.003 ^∘^	0.907 ± 0.003 ^∘^	0.454 ± 0.009	0.354 ± 0.007
Wiener (point)	0.474 ± 0.003 ^∘^	0.908 ± 0.002	0.498 ± 0.006	0.431 ± 0.005
Wiener (belief)	0.475 ± 0.003 ^∘^	0.909 ± 0.002	0.496 ± 0.006	0.405 ± 0.005
LSTM (point)	0.475 ± 0.003 ^∘^	0.909 ± 0.002	0.490 ± 0.006	0.427 ± 0.006
Proposed	0.475 ± 0.003	0.907 ± 0.002	0.494 ± 0.006	0.437 ± 0.007
Genuine CSI (bound)	0.476 ± 0.003 ^∘^	0.915 ± 0.002	0.508 ± 0.006	0.402 ± 0.004

**Table 10:** Complexity of the added components (batch size one, float32, CPU; platform in Section 6.5).

Component	Parameters	Latency, 1 Thread Median [p10, p90]	Latency, 2 Threads Median
Semantic encoder $f_{\theta }$	157 k	1.18 ms [1.09 ms, 1.49 ms]	1.34 ms
Semantic decoder $g_{\phi }$	58 k	1.15 ms [1.07 ms, 1.39 ms]	1.31 ms
LSTM channel predictor	52 k	0.60 ms [0.54 ms, 0.70 ms]	0.66 ms
Risk-aware controller	table, 312 entries	71 μs [70 μs, 0.11 ms]	72 μs

## Data Availability

The sensor streams used in this study are publicly available through the Numenta Anomaly Benchmark [23,24], and the measured 5G/LTE channel logs are publicly available as the 5G production dataset of Raca et al. [25].

## Abbreviations

The following abbreviations are used in this manuscript:

ACE	Average coverage error
AWGN	Additive white Gaussian noise
CSI	Channel state information
IoT	Internet of things
JSCC	Joint source–channel coding
LMMSE	Linear minimum mean-squared error
LSTM	Long short-term memory
MIMO	Multiple-input multiple-output
NAB	Numenta Anomaly Benchmark
NMSE	Normalized mean-squared error
OFDM	Orthogonal frequency-division multiplexing
PADC	Predictive and adaptive deep coding
PICP	Prediction interval coverage probability
RMSE	Root-mean-squared error
SCMA	Sparse code multiple access
SNR	Signal-to-noise ratio
